# Coordination between the Ndc80 complex and dynein is essential for microtubule plus-end capture by kinetochores during early mitosis

**DOI:** 10.1016/j.jbc.2023.104711

**Published:** 2023-04-14

**Authors:** Mohammed Abdullahel Amin, Manas Chakraborty, Destiny Ariel Wallace, Dileep Varma

**Affiliations:** Department of Cell and Developmental Biology, Feinberg School of Medicine, Northwestern University, Chicago, Illinois, USA

**Keywords:** Mitosis, Ndc80, dynein, kinetochore, microtubule, attachment, capture, spindle, siRNA, CRISPR-Cas9, plus-end, kinetochore-null, *in vitro* reconstitution

## Abstract

Mitotic kinetochores are initially captured by dynamic microtubules via a “search-and-capture” mechanism. The microtubule motor, dynein, is critical for kinetochore capture as it has been shown to transport microtubule-attached chromosomes toward the spindle pole during prometaphase. The microtubule-binding nuclear division cycle 80 (Ndc80) complex that is recruited to kinetochores in prophase is known to play a central role in forming kinetochore-microtubule (kMT) attachments in metaphase. It is not yet clear, however, how Ndc80 contributes to initial kMT capture during prometaphase. Here, by combining CRISPR/Cas9-mediated knockout and RNAi technology with assays specific to study kMT capture, we show that mitotic cells lacking Ndc80 exhibit substantial defects in this function during prometaphase. Rescue experiments show that Ndc80 mutants deficient in microtubule-binding are unable to execute proper kMT capture. While cells inhibited of dynein alone are predominantly able to make initial kMT attachments, cells co-depleted of Ndc80 and dynein show severe defects in kMT capture. Further, we use an *in vitro* total internal reflection fluorescence microscopy assay to reconstitute microtubule capture events, which suggest that Ndc80 and dynein coordinate with each other for microtubule plus-end capture and that the phosphorylation status of Ndc80 is critical for productive kMT capture. A novel interaction between Ndc80 and dynein that we identify in prometaphase extracts might be critical for efficient plus-end capture. Thus, our studies, for the first time, identify a distinct event in the formation of initial kMT attachments, which is directly mediated by Ndc80 and in coordination with dynein is required for efficient kMT capture and chromosome alignment.

Sister kinetochores are captured by the dynamic spindle microtubules during early mitosis to form kinetochore-microtubule (kMT) attachments, an essential prerequisite for proper chromosome alignment and segregation. Kinetochores are large macromolecular machines assembled on the centromeric DNA that bridges sister chromatids to dynamic microtubules during mitosis and drive the accurate partitioning of the genetic material into the daughter cells. Over 100 proteins constitute the human kinetochores consisting of the inner kinetochore and the outer kinetochore regions (1, 2, 3, 4). While the inner kinetochore proteins connect with the centromeric DNA and form a platform for the assembly of the outer kinetochore, the outer kinetochore proteins are involved in microtubule binding (5, 6). Even though a large number of outer kinetochore proteins have been shown to bind to microtubules, it is not clear which of these proteins actually contribute to the initial capture of human kinetochores during early stages of mitosis. Moreover, the mechanistic details of how kinetochore protein(s) enable the capture of kinetochores by microtubules during early mitosis is still not understood.

During early mitosis, outer surface of unattached kinetochores transiently expands outward to form a fibrous corona and associate laterally with the sides of the microtubules (7, 8, 9, 10, 11, 12, 13). The capture of chromosomes during early mitosis is primarily thought to be initiated as lateral contacts of kinetochores with the sides of spindle-pole-nucleated microtubules followed by the transport of captured kinetochores along the microtubule, which, in yeast is promoted by a kinesin-14 family member kar3 (13, 14). Previous work suggests that the minus-end–directed microtubule motor, cytoplasmic dynein, is required for kinetochore capture, lateral attachment, and poleward motility in large cells such as newt pneumocytes (15). However, while succeeding work has supported dynein’s role in poleward motility *via* dynamic lateral contacts with spindle microtubules in smaller human cells in culture, it is not clear from these studies, the extent to which dynein function is required for the initial attachment of kinetochores to microtubules.

Among the other outer kinetochore proteins, the conserved nuclear division cycle 80 (Ndc80) complex consisting of Ndc80/highly expressed in cancer protein 1 (Hec1), Nuf2, Spc24, and Spc25 plays a key role in forming robust interactions with microtubules during metaphase (16, 17). The Ndc80 complex binds directly to microtubule polymers through a CH (calponin homology) domain and a positively charged, unstructured amino-terminal tail (18, 19, 20). While, no motor function was attributed to this complex, initial studies on the Ndc80 complex clearly indicated that it is required for kMT attachment and chromosome congression in different model systems (21, 22, 23, 24) However, further studies have focused on the function of this complex in forming robust end-on kMT attachments during metaphase and anaphase, and the role that the Ndc80 complex plays in initial kMT attachments have largely remained unexplored. A key reason for this is that further studies revealed a high levels of phosphorylation of the tail domain by mitotic kinase, Aurora B, being instrumental in reducing the affinity of Ndc80 for microtubules in prometaphase, while lower phosphorylation levels in metaphase and anaphase enhanced Ndc80 microtubule binding to promote robust kMT attachments (5, 16). Moreover, cells with disrupted Ndc80 complex function have severe defects in stabilizing kMT attachments in metaphase leading to extensive chromosome misalignment, mis-segregation (25, 26), or even completely fail to segregate the chromosomes (27, 28).In short, while the role of Ndc80 complex in the formation of stable kMT attachments has been studied intensively, it is not clear how the microtubule-binding function of this complex contributes to kMT attachment formation during early mitosis. Further, while it is evident that the Ndc80 complex needs to functionally coordinate with dynein for kMT capture and chromosome congression, the nature of cross-talk between these two complexes is not well understand (29).

Based on the previous research in this area, we reasoned that the process of kMT capture during early mitosis could be functionally separated into two steps: (i) the initial kMT attachment formation and (ii) the ensuing poleward transport of kinetochores, which is known to be dependent on dynein. Both these steps need to be successfully completed to constitute a productive kinetochore capture event and is a prerequisite for proper chromosome congression. Further, it is relevant to point out that the initial kMT attachments which are formed between dynamic microtubules and individual mono-oriented kinetochores are functionally distinct from the attachments that occur later in mitosis (during metaphase and anaphase), that is known to require the function of the Ndc80 complex. This is so because the initial kMT attachments are not load bearing and are prone to easy detachments (to facilitate kinetochore motility on microtubules). On the other hand, the attachments in metaphase/anaphase are load bearing (due to bi-attachment with microtubules of opposite spindle poles) and are stabilized with the help of protein factors that are accessory to Ndc80 (including the Ska1 complex, Cdt1, Astrin, etc.) to prevent kinetochore detachment during chromosome segregation (5, 30).

In this study, we specifically focus on the mechanism of the first step in kMT capture, *i.e.*, the formation of initial kMT attachments. By combining CRISPR/Cas9 gene editing and RNA interference (RNAi) technology with targeted assays employing high-resolution confocal microscopy, we find that the function of the Ndc80 complex is essential for the formation of initial attachment kinetochore by microtubules during early mitosis. Further, we find that the expression of Ndc80 mutants deficient in microtubule attachment are unable to rescue the defects in kMT capture observed in Ndc80-depleted cells. Interestingly, the inhibition of dynein however only disrupted a substantially smaller fraction of initial kMT attachment events as compared to Ndc80 inhibition. Our studies point to a scenario where the dynein motor and the Ndc80 complex synergize during early mitosis to facilitate microtubule-end capture and contribute to productive kinetochore capture events to drive chromosome congression.

## Results and discussion

### Depletion of Ndc80 complex causes defects in initial kMT attachments

**Figure 1:**
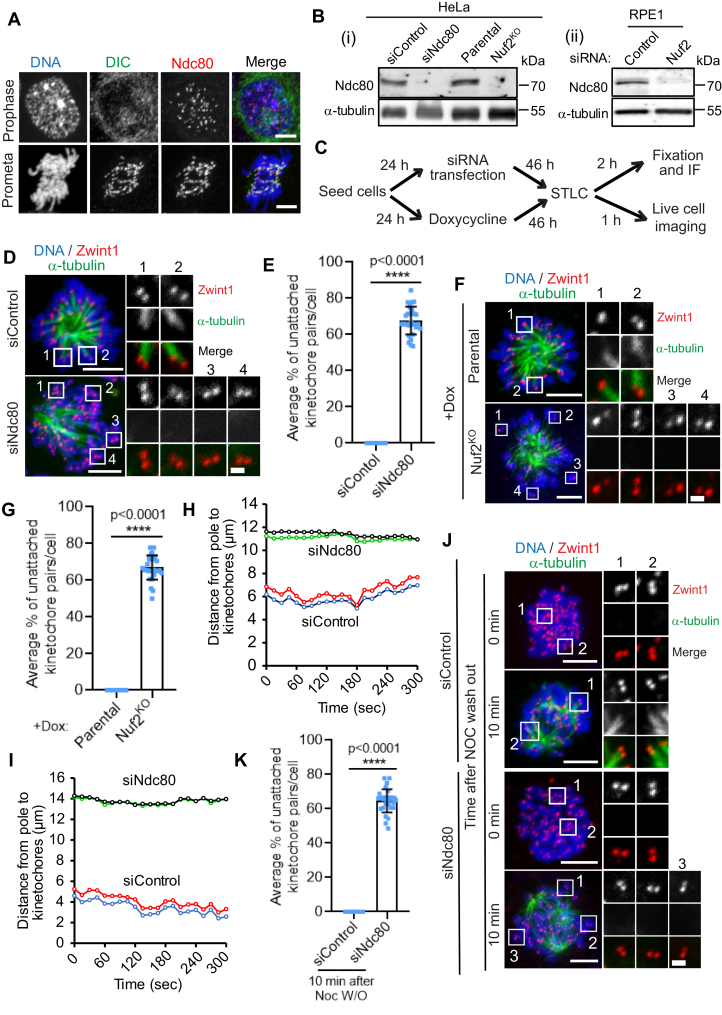
**Initial kMT attachments are defective after the inhibition of the Ndc80 complex.***A*, immunofluorescence staining of dynein intermediate chain (DIC, *green*) and Ndc80 (*red*), in early mitotic prophase (*top panels*) or prometaphase (*bottom panel*), with the chromosomes counterstained using DAPI. Bars, 5 μm. *B*, Western blot analysis of HeLa cells treated with siRNAs for control and Ndc80 (Bi) of HeLa cells of parental and Nuf2 CRIPR/Cas9 KO (*B*) and of RPE1 cells treated with siRNAs for control and Nuf2 (Bii). α-tubulin was used as a loading control. *C*, cells were subjected to the abovementioned perturbations, then treated with the indicated drugs/chemicals and fixed according to the scheme. *D* and *F*, immunofluorescence staining of STLC-treated mitotic HeLa cells depleted of Ndc80 (*D*) or knocked out of Nuf2 (*F*) in comparison to respective control^RNAi^ or parental control cells and stained for α-tubulin (*green*), a kinetochore marker Zwint1 (*red*) with the chromosomes counterstained using DAPI. Bars, 5 μm. Inset shows the kMT attachment status of individual kinetochore pairs in the conditions indicated. Bar (applies to all insets), 1 μm. *E* and *G*, quantification of the status of kMT attachments in cells from *D* and *F*. Error bars represent S.D. from three independent experiments. For each experiment, on average ∼20 kinetochore pairs from ten different monopolar cells were examined. ∗∗∗∗*p* < 0.0001 (Student’s *t* test). *H* and *I*, trajectories of two representative sister kinetochore pairs in HeLa cells transfected with siRNAs for control and Ndc80. Fifty kinetochore pairs were analyzed for each condition. Also see Videos S1 and S2. *J*, HeLa cells treated with control^RNAi^ or Ndc80^RNAi^ were fixed 0 or 10 min after nocodazole washout. The cells were then immunostained for α-tubulin (*green*), a kinetochore marker Zwint1 (*red*), with chromosomes counterstained using DAPI. Bars, 5 μm. Inset shows the kMT attachment status of individual kinetochore pairs at these two time points under either condition. Bar (applies to all insets), 1 μm. Also see Fig. S2*A*. *K*, quantification of the status of kMT attachments in cells from *J*. Error bars represent S.D. from three independent experiments. For each experiment, ten mitotic cells were examined. ∗∗∗∗*p* < 0.0001 (Student’s *t* test). kMT, kinetochore-microtubule; Ndc80, nuclear division cycle 80; STLC, S-Trityl-l-Cysteine.

First, we systematically analyzed the localization of the microtubule-binding Hec1 subunit of the Ndc80 complex during early mitosis. Our immunofluorescence data show that Ndc80 is recruited to kinetochores during prophase, when no discernable dynein localization can yet be detected at kinetochores (Fig. 1*A*, top panel; Fig. S1*A*) (19, 31, 32). Mitotic cells in prometaphase, however, showed strong localization of both dynein and Ndc80 to kinetochores as expected (Fig. 1*A*, bottom panel; Fig. S1*A*). We then disrupted the function of the Ndc80 complex either by depleting the Hec1 or Nuf2 subunit using siRNA-mediated knockdown or by knocking out the Nuf2 subunit using CRISPR/Cas9-mediated gene editing technology. Efficient loss of Ndc80 levels was validated by immunoblotting (Fig. 1*B*) as well as immunostaining analyses (Fig. S1*B*) for all the approaches we used for the functional perturbation of Ndc80.

To accurately test for the role of Ndc80 in the initial kMT attachment step, we choose to first resort to a method that simulates the kMT attachments formed in early mitosis during the ‘search and capture’ process that are mono-oriented and not load bearing in nature. We treated cells with a small molecule S-Trityl-l-Cysteine (STLC), which inhibits the kinesin, Eg5, and mitotic spindle bipolarity to produce monopolar spindles (Fig. 1*C*) (33, 34). We observed that STLC-treated monopolar cells depleted of the Hec1 subunit of the Ndc80 complex (Ndc80^RNAi^) had significantly higher number of unattached kinetochore pairs compared to that of cells treated with control siRNA (control^RNAi^) and STLC. Careful analysis revealed that as compared to control cells where almost all kinetochore pairs had at least one kinetochore attached to the microtubules, ∼70% kinetochore pairs in Ndc80^RNAi^ cells were completely unattached, among all discernable kinetochore microtubule interaction events analyzed (Fig. 1, *D* and *E*). We then analyzed the percentage of unattached *versus* single kinetochore-attached (monotelic) *versus* both kinetochores of a kinetochore pair attached (syntelic) in control^RNAi^ and Ndc80^RNAi^ cells treated with STLC. In control STLC-treated cells, more than 95% kinetochores were syntelic. In contrast, in Ndc80^RNAi^ while 65 to 70% were unattached as mentioned previously, the remaining 25 to 30% captured kinetochore pairs were monotelic (Fig. S1*C*).We also observed that STLC-treated HeLa cells knocked out of Nuf2 (Nuf2^KO^) had a similar, significantly higher fraction (>65%) of kinetochore pairs which do not attach to microtubules as compared to that of parental cells (Fig. 1, *F* and *G*). Similar defects in initial kMT attachments (∼65% unattached) were observed in diploid RPE1 cells treated with STLC and depleted of Nuf2 (Nuf2^RNAi^) as compared to control^RNAi^ cells (Fig. S1, *D* and *E*). To further examine the initial kMT attachment events, we analyzed the dynamic oscillation of sister kinetochore pairs in STLC-treated cells by live-cell imaging. Our live imaging data showed that kinetochores in control^RNAi^ cells have robust oscillatory motion as expected as they were attached properly to the ends of dynamic kMTs (Fig. 1, *H* and *I*, siControl traces, Video S1). In contrast, kinetochores in Ndc80^RNAi^ cells showed remarkably lower incidence of oscillatory motion, undergoing very short-range, erratic or random, back-and-forth motion instead (Fig. 1, *H* and *I*, siNdc80 traces, Video S2).

### Other kinetochore microtubule-binding proteins play a less significant role in initial kMT attachment events as compared to Ndc80

As chromosomes are captured at the interface of outer kinetochores with microtubules, we next investigated the role of other outer microtubule binding and accessory kinetochore proteins known to have a role in proper chromosome alignment, including the dynein-dynactin complex, Spindly, CAP-GLY domain containing linker protein 170 (CLIP-170), and centrosome-associated protein E (CENP-E) (35, 36, 37, 38, 39, 40). Dynein, CLIP-170, and CENP-E have been proposed to generate lateral interactions between kinetochores and microtubules to aid in bi-directional chromosome movements during their congression to the metaphase plate (29, 41, 42). Spindly has been reported to be involved in rapid integration of peripheral chromosomes into the mitotic spindle but not as such in chromosome attachment to microtubules (43). Moreover, Spindly is involved in the prevention of load-bearing attachment formation during prometaphase in *Caenorhabditis elegans* (37). We first confirmed the localization to these proteins to prometaphase kinetochores during early mitosis (Fig. S2*A*). Next, we disrupted their function by treating with specific siRNAs and employing published protocols (44, 45, 46). We confirmed the efficiency of depletion of the target proteins by immunoblotting (Fig. S2*B*). We then tested if these outer kinetochore proteins have a role in initial kMT attachments by carefully visualizing these attachment events using the same STLC-treatment assay as we did for the Ndc80 inhibition experiments. Our data show that initial kMT attachments in STLC-treated cells depleted of dynein, p150glued (a dynactin subunit), CLIP-170, Spindly, CENP-E, or Knl1 was surprisingly normal as observed in control^RNAi^ cells (Fig. S2, *C* and *D*). However, in cells depleted of all of the abovementioned proteins (especially dynein and its accessory proteins), the chromosomes remained tethered to the plus-ends of microtubules constituting the monopolar spindles, consistent with previous work (47). These data suggest that most of the outer kinetochore proteins we tested, apart from the Ndc80 complex, do not play a substantial role in forming initial kMT attachments during early mitosis but rather could be involved in other mitotic functions, including chromosome congression or the spindle assembly checkpoint. As described earlier, the inhibition of the Ndc80 complex on the other hand severely compromised the initial step in kMT capture, *i.e.*, the formation of initial kMT attachments, yielding the more severe phenotype in defective kMT capture.

Our data support the previous observations that dynein motor activity is critical for the second step in the kMT capture process, *i.e.*, the poleward motility of the kinetochores, which ensues immediately after the formation of initial kMT attachments during early mitosis (15, 29, 47). Dynein-mediated lateral contacts are currently also understood to be the primary mode of initial kMT attachment formation. However, our observations along with the ability of the Ndc80 complex to facilitate load-bearing, end-on kMT attachments in metaphase that is well-established suggest that a considerable fraction of initial kMT attachment events in cultured human cells during early mitosis could also possibly be an outcome of dynamic end-on contact of microtubule plus-ends with individual kinetochores during the ‘search and capture’ process, rather than predominantly *via* lateral contacts as is commonly conceived. We propose that these dynamic end-on kMT attachments during early mitosis are mediated by the activity of the Ndc80 complex but with the key distinction that these attachments are not stabilized (and the kinetochore pairs are not bioriented) as in metaphase cells. Our findings also support the observations from previous studies that only minor defects in chromosome congression is observed in dynein or CENP-E depleted cells (8, 47, 48, 49), where only dynamic lateral initial kMT contacts are prevented. We predict that these lateral attachments, mediated by the abovementioned kinetochore motors, only account for ∼30% of all initial attachment events. Further, our results suggest that the loss of function of these kinetochore motor proteins could be compensated for by the activity of the Ndc80 complex over time to produce nearly normal chromosome alignment. Future live imaging studies of early mitotic cells with higher optical and temporal resolution are required to determine the fraction of initial kMT attachments facilitated by the Ndc80 complex that are either lateral or dynamic end-on in nature.

### *De novo* microtubules do not attach to kinetochores in Ndc80-depleted cells

*De novo* microtubules can be generated by treating cells with nocodazole to completely disassemble the cellular microtubules followed by washing out the drug and releasing into fresh culture medium (50, 51). We further investigated the role of Ndc80 in the initial attachment of kinetochores to *de novo* microtubules using the nocodazole washout assay (Fig. S3*A*). Nocodazole completely disassembles all the cellular microtubules; consequently, we found that HeLa cells treated either with Ndc80^RNAi^ (Fig. 1, *J* and *K*) or subjected to Nuf2^KO^ (Fig. S3, *B* and *C*) had no cellular microtubules at 0 mins after nocodazole washout similar to their respective controls. After 10 min of nocodazole washout, most of the kinetochores in control^RNAi^ HeLa cells or control parental HeLa cells were found to be attached to *de novo* microtubules. We insinuate that these microtubules could either be emanating from the centrosome (to make contact with the kinetochores later) or could directly be originating from the kinetochores themselves (52, 53). In contrast, a significant number (∼65%) of kinetochores were found to be not attached to *de novo* microtubules in Ndc80^RNAi^ or Nuf2^KO^ cells (Figs 1, *J* and *K* and S3, *B* and *C*). We also observed similar defects in initial kMT attachments in Nuf2^RNAi^ RPE1 cells as compared to control^RNAi^ cells. Nuf2^RNAi^ RPE1 cells had a significant number (∼65%) of kinetochores, which were unattached to the *de novo* microtubules 10 min after nocodazole washout compared to that in control^RNAi^ cells (Fig. S3, *D* and *E*). These data further indicate that kinetochores are not able to attach to newly formed microtubules in the absence of the Ndc80 complex during early mitosis. Interestingly, these experiments also suggest that the Ndc80 complex could contribute to the formation of initial kMT attachments by two mechanisms. First, the function of the complex could be required for directly forming lateral or end-on dynamic contacts of kinetochores with spindle-pole focused microtubules. Secondly, the microtubule-binding activity of Ndc80 could be critical for *de novo* kMT formation from kinetochores.

### Depletion of Ndc80 complex causes defects in the formation of initial kMT attachments in physiological conditions

**Figure 2:**
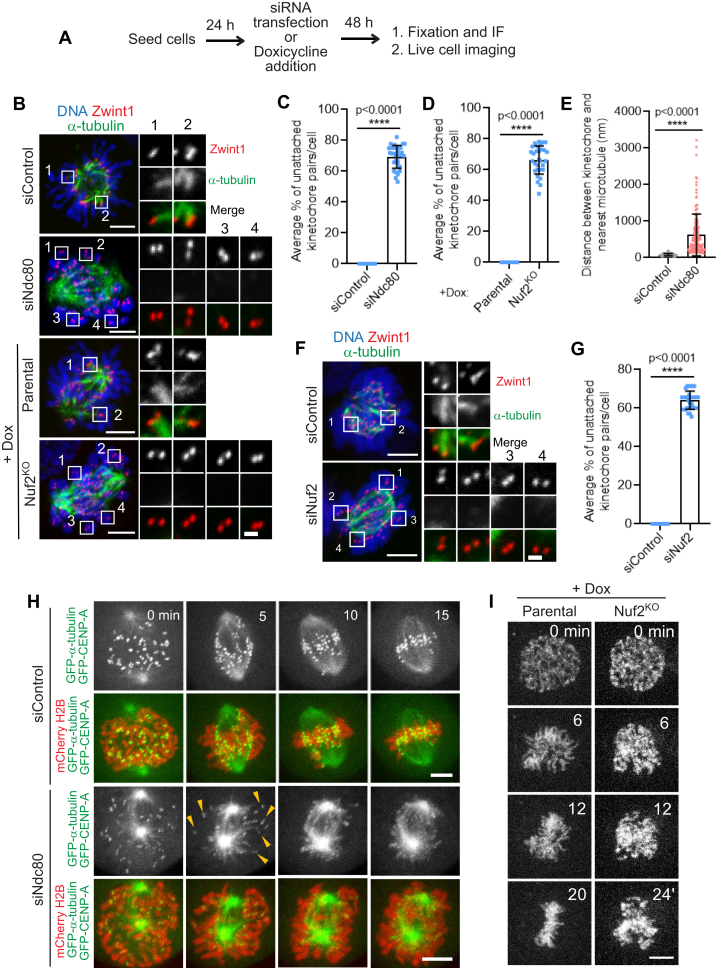
**Role of Ndc80 complex in kMT capture during early mitosis examined using fixed and live-cell imaging under physiological conditions.***A*, cells were subjected to the indicated perturbations treated with the indicated drugs/chemicals and fixed according to the scheme. *B*, immunofluorescence staining of early prometaphase HeLa cells depleted of Ndc80 (*top two panels*) or knocked out of Nuf2 (*bottom two panels*) in comparison to control^RNAi^ or parental cells, respectively, and stained for α-tubulin (*green*) and a kinetochore marker, Zwint1 (*red*) with the chromosomes counterstained using DAPI. Bars, 5 μm. Inset shows the kMT attachment status of individual kinetochore pairs in the indicated conditions. Bar (applies to all insets), 1 μm. *C* and *D*, quantification of the status of kMT attachments in cells from *B*. Error bars represent. S.D. from three independent experiments. For each experiment, on average ∼20 kinetochore pairs from ten early prometaphase cells were examined. ∗∗∗∗*p* < 0.0001 (Student’s *t* test). *E*, the distance measured between kinetochore and nearest microtubule in cells from *B*. Error bars represent S.D. from three independent experiments. For each experiment, on average 20 kinetochore pairs from five early prometaphase cells were examined. ∗∗∗∗*p* < 0.0001 (Student’s *t* test). *F*, immunofluorescence staining of early prometaphase RPE1 cells depleted of Nuf2 in comparison to control^RNAi^ cells (*top panel*) and stained for α-tubulin (*green*), a kinetochore marker Zwint1 (*red*) with the chromosomes counterstained using DAPI. Bars, 5 μm. Inset shows the kMT attachment status of individual kinetochore pairs. Bar (applies to all insets), 1 μm. *G*, quantification of the status of kMT attachments in cells from *F*. Error bars represent S.D. from three independent experiments. For each experiment, on average ∼20 kinetochore pairs from ten early prometaphase cells were examined. ∗∗∗∗*p* < 0.0001 (Student’s *t* test). *H*, selected frames from live imaging of double thymidine–synchronized HeLa cells stably expressing mCherry-Histone H2B to visualize the chromosomes, in addition to GFP-α-tubulin and GFP-CENPA to visualize kMT attachment and treated with control^RNAi^ (*top two panels*) or Ndc80^RNAi^ cells (*bottom two panels*). Images were captured every 1 min interval for 15 to 20 min until chromosome is aligned in control^RNAi^. Bars, 5 μm. *Yellow arrowheads* indicate the kinetochores unattached to microtubule in Ndc80^RNAi^ cells. The images from the videos were cropped and image intensity adjusted as required for better visualization of kinetochores and microtubules. *I*, selected frames from live imaging of parental (*left panel*) and Nuf2^KO^ (*right panel*) HeLa cells treated with Hoechst for 30 min prior to imaging to visualize the chromosomes. Images were captured every 2 min interval for around 30 min until all the chromosomes were aligned in parental cells. Bars, 5 μm. kMT, kinetochore-microtubule; Ndc80, nuclear division cycle 80.

Having already employed two distinct assay conditions to monitor initial kMT attachment formation in Ndc80-inhibited cells, we next tested the status of these events under normal physiological conditions by using both fixed and live-cell imaging (Fig. 2*A*). Our fixed cell analysis showed that Ndc80^RNAi^ HeLa cells had significantly higher number (>65%) of kinetochore pairs which are unattached to microtubules during early mitosis compared to that of control^RNAi^ cells (Fig. 2, *B* and *C*). Next, we quantified the distance of separation between kinetochore and the nearest microtubule loci (attached or unattached) within the limits of our confocal microscopy to better define the status of the initial kMT attachments. We observed that the average kMT distance was ∼85 nm when kinetochores are attached to microtubules in control^RNAi^ cells (see Methods for more information). On the other hand, in Ndc80^RNAi^ cells, while there were no microtubules for distances >1 μm within the vicinity for many kinetochores, the average kMT distance was found to be ∼750 nm in cases where a microtubule was observed to be in the vicinity of a kinetochore (Fig. 2*E*). We found that Nuf2^KO^ HeLa cells also had >65% of their kinetochore pairs not captured by microtubules in early mitosis as compared to that of parental cells (Fig. 2, *B* and *D*). Besides HeLa cells, we observed similar defects (∼65%) in initial kMT attachments in Nuf2^RNAi^ RPE1 cells (Fig. 2, *F* and *G*).

We further confirmed the defects in initial kMT attachments in Ndc80-inhibited cells under normal physiological conditions by observing kMT behavior during early mitosis in live cells. Previous studies using both fixed and live-imaging experiments (54, 55, 56) suggest that mitotic cells are unable to align a large fraction of their chromosomes for an extended period of time after Ndc80 inhibition. Apart from visualizing kMT behavior, the labeling of spindle microtubule in our live experiments also ensured that bipolar spindles are formed normally in Ndc80-inhibited cells. To ensure that the observed phenotype was not accumulated over several cell cycles, we chose to use double-thymidine synchronized HeLa cells, where Ndc80 knockdown was performed only during the two successive periods of thymidine treatment, for our live imaging (54, 57). Our studies show that in control^RNAi^ cells, all the kinetochores are attached to microtubules within 5 to 10 min after nuclear envelope breakdown and are completely aligned at the metaphase plate within 15 to 20 min (Fig. 2*H*, top two panels, Video S3). In contrast, a substantial number of sister kinetochore pairs (∼65%) of Ndc80^RNAi^ cells remained unattached to microtubules and were not able to align properly at the metaphase plate in the same duration of time (Fig. 2*H*, bottom two panels, Video S4). Live-cell imaging of Nuf2^KO^ HeLa cells with their DNA labeled using Hoechst further shows that chromosome alignment is severely impeded in this scenario as compared to parental controls supporting the live imaging results obtained with Ndc80^RNAi^ synchronized HeLa cells (Fig. 2*I*; Videos S5 and S6).

### Ndc80’s role in initial kMT attachment is dependent on its ability to bind microtubules

**Figure 3:**
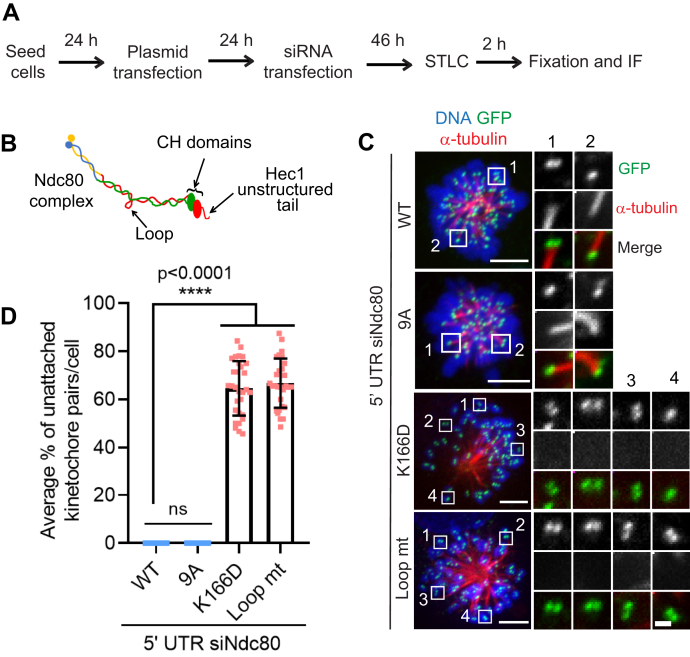
**Characterization of different microtubule-binding domains within the Ndc80 complex required for the initial kMT attachments.***A*, cells were subjected to the indicated perturbations, treated with the indicated drugs/chemicals, and fixed according to the scheme. *B*, a cartoon showing the Ndc80 complex with different domains of the Hec1 subunit that have been implicated in kinetochore-microtubule (kMT) attachments. *C*, immunofluorescence staining of STLC-treated mitotic HeLa cells depleted of endogenous Ndc80 and transfected with different Hec1-GFP constructs as indicated and stained for GFP (*green*), α-tubulin (*red*) with the chromosomes counterstained using DAPI. Bars, 5 μm. Inset shows the kMT attachment status of individual kinetochore pairs in the conditions indicated. Bar (applies to all insets), 1 μm. *D*, quantification of the status of kMT attachments in cells from *C*. Error bars represent S.D. from three independent experiments. For each experiment, on average ∼20 kinetochore pairs from ten monopolar cells were examined. ∗∗∗∗*p* < 0.0001 (Student’s *t* test). Ndc80, nuclear division cycle 80; STLC, S-Trityl-l-Cysteine.

To obtain additional insight into the role played by Ndc80 in initial kMT attachments, we performed rescue experiments using different Ndc80 mutants that have been shown to be defective in forming load-bearing kMT attachments in metaphase. The microtubule-binding activity of the Ndc80 complex is known to be located at two distinct elements within the amino terminus of its Hec1 subunit: a positively charged, unstructured amino-terminal tail with Aurora B phosphorylation sites dispersed within this region and the adjacent CH domain, a globular microtubule-binding domain found in proteins (Fig. 3*B*) (18, 55, 58, 59, 60, 61). High-resolution cryo-EM and *in vitro* analyses has further shown that both the N-terminal tail and CH domains are important for efficiently binding to microtubules (20, 56, 58, 62, 63, 64, 65, 66). In cells, the N-terminal tail and CH domain of Hec1 have been reported to be critical to form stable kMT attachments (55, 56, 67). Rescue experiments have shown that the stability of kMT attachments is affected by the phosphorylation state of the N-terminal tail domain of Hec1. Cells expressing a nonphosphorylatable Hec1 tail domain (exhibited hyperstable kMT attachments leading to premature chromosome segregation (59, 67, 68).

We first tested whether the expression of the N-terminal nonphosphorylatable mutant of Hec1 could rescue initial kMT attachment formation using the indicated experimental scheme (Fig. 3*A*). Our rescue experiments showed that Ndc80^RNAi^ cells transfected with Hec1^WT^ or nonphorylatable Hec1^9A^ mutant constructs and treated with STLC had no observable defects in the formation of early kMT attachments as expected (Fig. 3, *C* and *D*). As mentioned, mutations in its CH domain have been reported to severely impair Ndc80’s microtubule binding *in vitro* and the formation of stable kMT attachment *in vivo* due to a change in protein conformation that compromises this activity (56, 58, 64, 65). Thus, we next tested whether role of Ndc80 in initial kMT attachments is influenced by the CH domain mutant (Hec1^K166D^), defective in microtubule binding. Our rescue experiments showed that Ndc80^RNAi^ cells transfected with the Hec1^K166D^ mutant construct and treated with STLC also had a significant fraction (∼65%) of kinetochores which do not attach to microtubules as compared to cells rescued with Hec1^WT^ (Fig. 3, *C* and *D*). These data suggest that CH domain-mediated physiological conformation of Hec1 is also essential for initial kMT capture. In addition to the established microtubule-binding sites within the Ndc80 complex, the internally located loop domain has also been reported to be important for the recruitment of Ndc80 accessory proteins such as the Ska complex and Cdt1 to human kinetochores, where they are required to form end-on kMT attachments during metaphase (30, 69). We hence used the STLC treatment assay to test if the Hec1 loop domain was required for kMT capture. Surprisingly, we find that rescue experiments with a Hec1 loop domain mutant (Hec1^LoopMut^) also exhibited similar defects in kinetochore capture as observed with the other mutants defective in direct microtubule binding (Fig. 3, *C* and *D*). Further studies are required to define the mechanism by which the loop domain contributes to initial kMT attachments during early mitosis. Taken together, our data so far suggest that the formation of initial kMT attachments mediated by Ndc80 is influenced by perturbations of the structural and regulatory components that control Ndc80-microtubule binding.

### Depletion of the Ndc80 complex leads to kinetochore-null phenotype when Aurora B kinase is inhibited simultaneously

**Figure 4:**
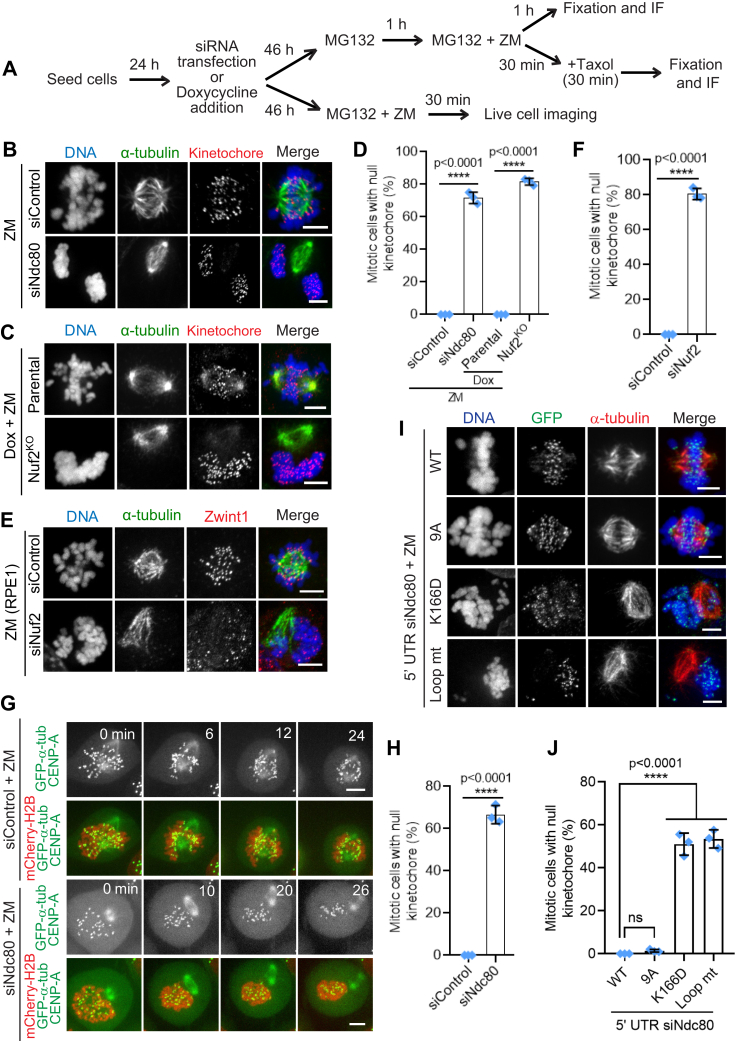
**Characterization of the kinetochore-null phenotype induced by Ndc80 inhibition.***A*, cells were subjected to the indicated perturbations, treated with the indicated drugs/chemicals, and fixed according to the scheme. *B* and *C*, mitotic HeLa cells depleted of Ndc80 (*B*, panel) or knocked out of Nuf2 (*C bottom panel*) as compared to control^RNAi^ (*B*, *top panel*) or parental control cells (*B*, *bottom panel*), and followed by the indicated drug treatments, were immunostained for α-tubulin (*green*), a kinetochore marker Zwint1 or CENPA (*red*) with the chromosomes counterstained using DAPI. Bars, 5 μm. *D*, quantification of the frequency of mitotic cells with null kinetochores (see main text for more details) in samples from *B* and *D*. Error bars represent S.D. from three independent experiments. For each experiment, 200 mitotic cells were examined. ∗∗∗∗*p* < 0.0001 (Student’s *t* test). *E*, immunofluorescence staining of mitotic RPE1 cells depleted of Nuf2 in comparison to control cells (*top panel*) and stained for α-tubulin (*green*), a kinetochore marker Zwint1 (*red*) with the chromosomes counterstained using DAPI. Bars, 5 μm. *F*, quantification of the status of kMT attachments in cells from *E*. Error bars represent S.D. from three independent experiments. For each experiment, 200 mitotic cells were examined. ∗∗∗∗*p* < 0.0001 (Student’s *t* test). *G*, selected frames from live imaging of HeLa cells stably expressing mCherry-Histone H2B to visualize the chromosomes in addition to GFP-α-tubulin and GFP-CENPA to visualize kMT attachment in control^RNAi^ (*top two panels*) and Ndc80^RNAi^ cells (*bottom two panels*). Images were captured at every 1 min interval starting from the point of nuclear envelope breakdown for around 30 min until a proper mitotic spindle was formed during prometaphase in control^RNAi^ cells. Bars, 5 μm. *H*, quantification of mitotic cells with null kinetochores from *G*. Error bars represent S.D. from three independent experiments. For each experiment, 50 mitotic cells were examined. *I*, immunofluorescence staining of MG132 + ZM treated mitotic HeLa cells depleted of endogenous Ndc80 and transfected with different Hec1-GFP constructs as indicated and stained for GFP (*green*), α-tubulin (*red*) with the chromosomes counterstained using DAPI. Bars, 5 μm. *J*, quantification of mitotic cells with null kinetochores from *A*. Error bars represent S.D. from three independent experiments. For each experiment, 200 mitotic cells were examined. ∗∗∗∗*p* < 0.0001 (Student’s *t* test). kMT, kinetochore-microtubule; Ndc80, nuclear division cycle 80; ZM, ZM447439.

kMT attachments are regulated by Aurora B kinase-mediated variable phosphorylation of the Ndc80 complex during the different stages of mitosis (18, 59, 70). While Aurora B destabilizes kMT contacts during early mitosis to aid in attachment error correction, inhibition of the kinase activity using small-molecule inhibitors leads to premature stabilization of kMT attachments of nonbioriented prometaphase kinetochores (71, 72, 73). We thus reasoned that hampering Aurora B activity using the single molecule inhibitor, ZM447439 (henceforth referred to as ZM) (using the indicated protocol, Fig. 4*A*), should rescue the defects in initial kMT attachments observed in Ndc80-inhibited cells during early mitosis. For our experiments, we coupled the inhibition of Aurora B with MG132 treatment to prevent premature anaphase onset. We found that control^RNAi^ or parental mitotic HeLa cells attained partial chromosome alignment, presumably because the formation of initial kMT attachments occurred normally as expected in these cells (Fig. 4, *B* and *C*, top panels, Fig. 4*D*). Surprisingly, after ZM treatment, kinetochores of around 75% mitotic Ndc80^RNAi^ or Nuf2^KO^ cells were found completely outside of the mitotic spindle, uncoupled from the microtubules, a phenomenon previously referred to as the “kinetochore-null” phenotype (37, 74) (Fig. 4, *B* and *C*, bottom panels, Fig. 4*D*). We then asked whether the kinetochore-null phenotype is produced under similar conditions but even in the presence of paclitaxel (Taxol), a small molecule that stabilizes microtubules in general, thus also serving to rescue the combined effects of Aurora B and Ndc80 inhibition, at least partially. We found that a significant number of mitotic Ndc80^RNAi^ or Nuf2^KO^ (Fig. S4, *A* and *B*, bottom panels; Fig. S4*C*) cells continued to exhibit the kinetochore-null phenotype even in this scenario (ZM + Taxol) as compared to control^RNAi^ cells or parental cells (Fig. S4, *A* and *B*, top panels; Fig. S4*C*). Further, as observed in HeLa cells, 75% of mitotic Nuf2^RNAi^ RPE1 cells also showed the kinetochore-null phenotype which was significantly higher as compared to that of control^RNAi^ cells after the inhibition of Aurora B (Fig. 4, *E* and *F*). We then analyzed this interesting kinetochore-null phenotype using live-cell imaging. No control^RNAi^ cells showed this phenotype in the presence of ZM during the period (∼25 min) of our live imaging, suggesting that kinetochore capture and alignment is normal in this case (Fig. 4, *G* and *H*, top two panels, Video S7). In contrast, a significantly higher portion (∼70%) of mitotic Ndc80^RNAi^ cells showed the kinetochore-null phenotype (Fig. 4, *G* and *H*, bottom two panels, Video S8) where kinetochores (marked by GFP-fused CENP-A) were observed to remain completely separated away from the vicinity of the mitotic spindle during the entire period of our live imaging procedure, without initiating any capture events.

We then analyzed if the microtubule-binding mutants of Ndc80 could rescue the kinetochore-null phenotype produced in Ndc80^RNAi^ cells after ZM treatment. Our immunostaining data showed that cells rescued with Hec1^WT^ or nonphorylatable Hec1^9A^ mutant constructs rarely produced the kinetochore-null phenotype when Aurora B was inhibited (Fig. 4, *I* and *J*, top two panels). In contrast, cells rescued with Hec1^K166D^ or Hec1^LoopMut^ constructs showed a significantly high frequency of kinetochore-null phenotype in the same condition (Fig. 4, *I* and *J*, bottom three panels as indicated). This finding further supports the notion that the microtubule-binding activity of Ndc80 is directly responsible for the formation of initial kMT attachments during early mitosis.

### The Ndc80 complex coordinates with the dynein motor for effective kinetochore capture

**Figure 5:**
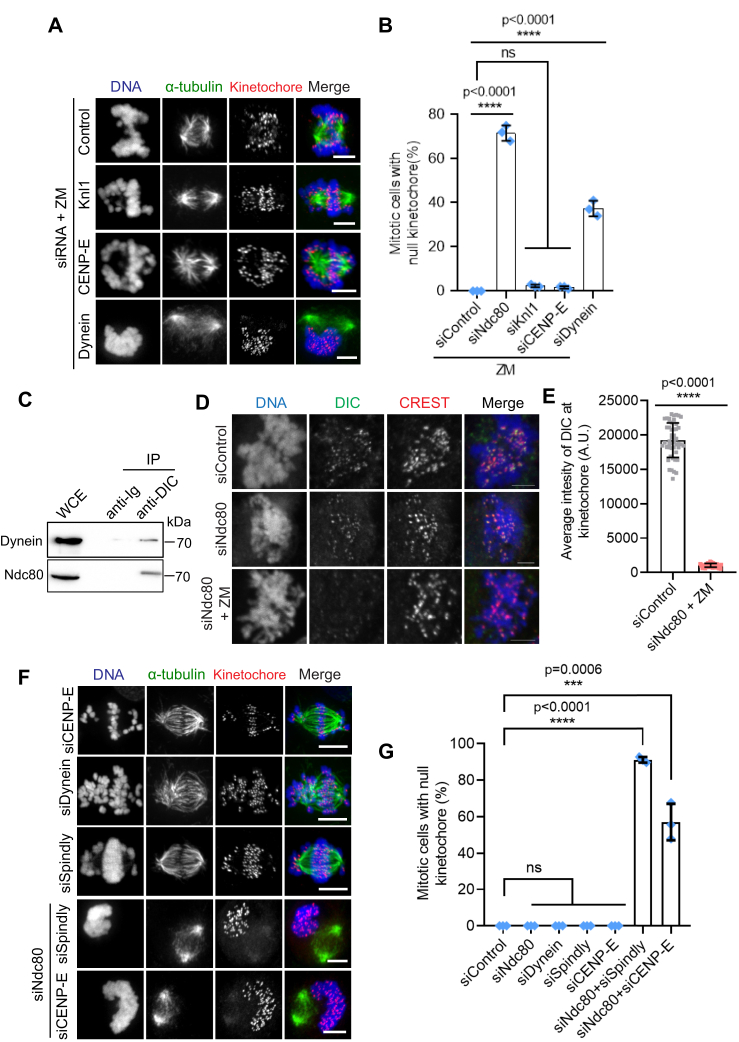
**The Ndc80 complex coordinates with dynein for efficient kMT capture.***A*, immunofluorescence staining of MG132 + ZM treated mitotic HeLa cells depleted of the indicated proteins and stained for α-tubulin (*green*), a kinetochore marker (Zwint1 or CENP-A, *red*) with the chromosomes counterstained using DAPI. Bars, 5 μm. *B*, quantification of mitotic cells with null kinetochores from *A*. Error bars represent S.D. from three independent experiments. For each experiment, 200 mitotic cells were examined. ∗∗∗∗*p* < 0.0001 (Student’s *t* test). *C*, control (Ig) and anti-dynein IC immunoprecipitations (IPs) were performed using whole cell extracts prepared from nocodazole-treated prometaphase cells and the western blots of the IPs were probed with anti-dynein and anti-Ndc80 antibodies. *D*, analyses of dynein localization to kinetochores in Ndc80-depleted cells after Aurora B inhibition. Prometaphase HeLa cells were either treated with Ndc80 siRNA alone (*middle panel*) or in combination with ZM (*bottom panel*) as compared to untreated controls (*top panel*), followed by fixation and immunostaining for the dynein intermediate chain (DIC, *green*), a kinetochore marker (CREST, *red*) with the chromosomes counterstained using DAPI. Bars, 5 μm. *E*, quantification of dynein intensity for data in *D* for Ndc80-depleted cells after Aurora B inhibition as compared to the corresponding controls. A total of 50 kinetochores were analyzed from at least five different prometaphase cells. *F*, immunofluorescence staining of mitotic HeLa cells depleted of either CENP-E (*top panel*), dynein (*second panel*) or Spindly (*third panel*) alone or codepleted of either Ndc80 + Spindly (*fourth panel*) or Ndc80 + CENP-E (*bottom panel*) and stained for α-tubulin (*green*), a kinetochore marker (Zwint1, *red*) with the chromosomes counterstained using DAPI. Bars, 5 μm. *G*, quantification of mitotic cells with null kinetochores from *E*. Error bars represent S.D. from three independent experiments. For each experiment, 200 mitotic cells were examined. ∗∗∗∗*p* < 0.0001 (Student’s *t* test). CENP-E, centrosome-associated protein E; kMT, kinetochore-microtubule; Ndc80, nuclear division cycle 80.

In addition to the Ndc80 complex, we tested if a similar kinetochore-null phenotype could be observed after the inhibition of dynein, CENP-E, or Knl1, in combination with ZM treatment. Interestingly, our immunofluorescence imaging data show that cells depleted of dynein in combination with ZM treatment also showed a kinetochore null phenotype but to a significantly lower extend as compared to Ndc80^RNAi^ cells. On the other hand, cells depleted of CENP-E or Knl1 did not exhibit a significant kinetochore-null phenotype under similar conditions (Fig. 5, *A* and *B*).

The observation that the co-inhibition of Ndc80 and Aurora B resulted in a complete loss of initial kMT attachments was quite intriguing, and we wanted to explore this phenotype further. It has previously been shown that Aurora B kinase activity is required to recruit the microtubule motors, dynein and CENP-E that are responsible for chromosome congression to the kinetochores (70, 75, 76). Moreover, our previous findings showed that Ndc80 competes with dynein to bind microtubules (45). We first tested if there is a physical association of Ndc80 with the two kinetochore microtubule motors in early mitosis by an immunoprecipitation assay. Our experiments showed that Ndc80 indeed exhibited a novel interaction with dynein (Fig. 5*C*) but not with CENP-E (data not shown), in prometaphase cell extracts. This result along with the observation that CENP-E inhibition did not produce significant defects in initial kMT attachments by itself or in combination with ZM treatment, suggested to us that the kinetochore-null phenotype could be produced due to the loss of dynein recruitment to Ndc80-depleted kinetochores after Aurora B inhibition. To test this further, we assessed dynein localization to prometaphase kinetochores in Ndc80-depleted cells treated with ZM. While we did not observe a considerable reduction in the levels of dynein at Ndc80-depleted kinetochores (25) (Fig. 5*D*, top and middle panel), we found that dynein immunostaining was almost completely lost (>95%) when Ndc80-depletion was combined with Aurora B inhibition (Fig 5*D*, bottom panel; Fig. 5*E*).

To substantiate our results further, we performed codepletion experiments of Ndc80 with either dynein or CENP-E. Since we observed that the codepletion of the dynein motor and the Ndc80 complex was too toxic for the cells to tolerate, we depleted, Spindly, a recruiter of dynein to the kinetochore, in place of dynein, which retained the viability of the cells. Interestingly, we found the codepletion of Ndc80 and Spindly yielded an even higher frequency of the kinetochore-null phenotype (>90%) compared to that observed in cells co-inhibited of Ndc80 and Aurora B (∼75%) while single depletion of any of these proteins in itself did not yield this phenotype (Fig. 5, *B*, *F* and G). Codepletion of Ndc80 and CENP-E, on the other hand, also yielded a significant kinetochore-null phenotype than that observed with the depletion of either of the proteins individually but weaker than that observed for Ndc80+Aurora B or Ndc80+Spindly co-inhibition (Fig. 5, *F* and *G*, and data not shown). Taken together, these data suggest that productive kMT capture events during early mitosis (as described in the introduction) depends on the concerted function of the both the Ndc80 complex and the dynein motor.

### Ndc80 and dynein together efficiently captures microtubules by their plus-ends

**Figure 6:**
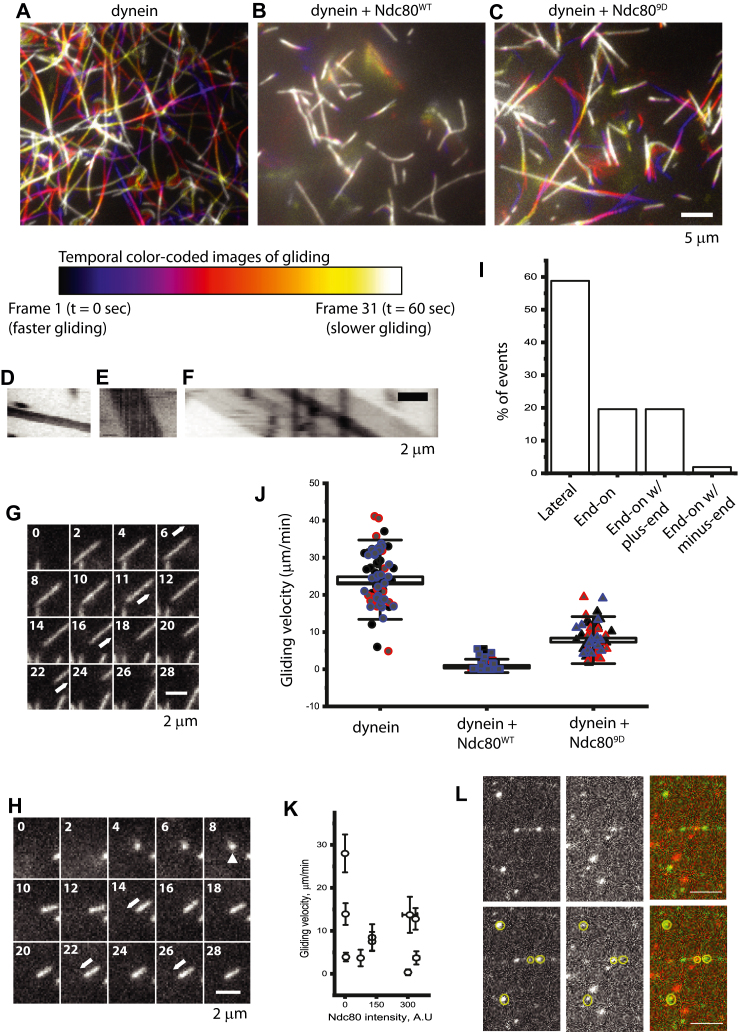
***In vitro* reconstitution of lateral and plus-end microtubule capture *via* the concerted function of dynein and the Ndc80 complex.***A*–*C*, temporal color-coded projections from a minute-long time lapse imaging of Taxol-stabilized microtubules (generated from Rhodamine-Tubulin) showing their gliding motility on the surface of immobilized dynein (*A*), dynein plus Ndc80^WT^-bonsai (*B*), or dynein plus Ndc80^9D^-bonsai (*C*). The *color-coded rectangle* below indicates the tapering-off of the microtubule-sliding velocity with the progress in time. Scale bar, 5 μm. *D*–*F*, representative kymographs, *D*, *E*, and *F* obtained from regions of designated microtubules from *A*–*C*, respectively, from above, demonstrating the velocity of sliding in each case. Scale bar, 2 μm, total duration of the kymographs, 1 min. *G* and *H*, montage of still images from time lapse videos of microtubule capture events in *C* from above (dynein + Ndc80^9D^-GFP) showing (*G*) lateral capture followed by sliding or (*H*) plus-end capture followed by sliding. *Arrows* indicate the direction of gliding, and the *arrowhead* in *H* indicates an end-on landing event. Scale bar, 2 μm; Time stamps are in s. *I*, quantification (percentage) of the various kinds of microtubules landing behavior with dynein plus Ndc80 (consolidated numbers for Ndc80 ^9D and WT^ -bonsai: *B* and *C* from above) immobilized on the surface. n = 51 landing events from N = 3 independent trials. *J*, box plot showing gliding velocity for the indicated conditions *A*–*C* from above. N = 3 independent trials, n ≥ 58 measurements. *K*, gliding velocity *versus* surface density of Ndc80^alexa647^ n ≥ 26 measurements for each point. Data = mean ± SEM. *L*, montage showing single molecules of dynein^alexa488^ (*left*, concentration 1 nM), Ndc80^alexa647^ (*center*, concentration 1 nM) and merge (*right*) bound to Taxol-stabilized microtubule (not imaged). *Bottom panel* showing exact same images, but *circles* indicate colocalized spots. Bar is 5 μm. Ndc80, nuclear division cycle 80.

In order to obtain a better understanding of how the Ndc80 complex and dynein co-operate together in the initial kMT attachment process, we reconstituted the capture event *in vitro* using purified proteins and total internal reflection fluorescence microscopy (TIR-FM). In our TIR-FM assay, we combined GFP-tagged Ndc80 (bonsai) complex comprising either of the Hec1^WT^ subunit (Ndc80^WT^-GFP) or its phosphomimetic version (Ndc80^9D^-GFP) together with the purified human whole dynein complex (Fig. S5*A*) (77). We expected the Ndc80^9D^-GFP to mimic the Aurora B-phosphorylated version of Ndc80 cells *in vitro*. Consistently, we find that Ndc80^9D^-GFP still retained considerable ability to bind Taxol-stabilized microtubules, which might favor dynamic attachments required for capture (Fig. S5*B*) (78). We reconstituted kinetochore-like microtubule capture with the help of a TIR-FM microtubule gliding assay over a lawn of either dynein alone or dynein together with the above mentioned versions of the Ndc80 complex (Fig. 6). We were able to clearly observe microtubules landing events on the surface where the proteins are immobilized. With only dynein immobilized, we only observed lateral landing events, where the entire body of the microtubule landed laterally on the surface followed by their directional gliding as expected (Fig. 6*A*, kymograph in D; Video S9). Intriguingly, when dynein was mixed with Ndc80 (both -WT and -9D), we also observed a substantial number of microtubule end-on landing events in addition to lateral attachment events (Fig. 6, *B* and *C*; kymographs in 6E, F; Video S9). While roughly, about 58% of landing events were lateral (Fig. 6, *G* and *I*; Videos S10–S12), about 42% of landing events were end-on (Fig. 6, *H* and *I*; Videos S10, S11 and S13). From among the 42% of the microtubules that landed end-on, 20% did not move any further, another 20% exhibited gliding events with plus-end leading forward and only about 2% exhibited gliding events with minus-end leading forward (Fig. 6*I*). A more detailed distribution based on individual trials is provided in Fig. S6*B*. This direction of movement clearly indicated that the microtubules almost exclusively landed with their plus-ends and were subsequently moved by dynein. Therefore, these landing events observed *in vitro* suggest that dynein and Ndc80 co-ordinate to directly capture microtubule plus-ends.

Our observations also indicate that there is a reduction in the number of microtubules bound to the surface of dynein/Ndc80 mixture compared to dynein alone (Fig. S6*C*). Further, we noticed that the gliding motion of microtubules in the presence of dynein and Ndc80^WT^ were severely impeded with an approximately 8-fold reduction in the microtubule gliding velocity (Fig. 6, *E* and *J*). This is in agreement with our previous work where we demonstrated that dynein microtubule binding could be inhibited by an excess of Ndc80 (45). On the other hand, we found that the phosphomimetic Ndc80^9D^-GFP rescued the microtubule binding density and that the microtubule gliding velocity was also recovered to >half the level of that observed with dynein alone (Fig. 6, *F* and *J*). Additionally, using fluorescent versions of both Ndc80 (full-length, see methods) and dynein, we were able to perform a titration experiment with variable surface density of Ndc80 (Fig. S6*A*-right). This titration experiment revealed that Ndc80 reduces dynein driven gliding motility in a stepwise manner (Fig. 6*K*). This reduction of gliding velocity of dynein by Ndc80 is not due to the reduction in the concentration of dynein as we found that the surface density of dynein^488^ remained unchanged (Fig. S6*A*-left). This suggests that the effect of Ndc80 on dynein is more than simple frictional resistance and is likely due to a more specific interaction. To test this idea further, we performed single molecule microtubule binding colocalization experiments to investigate whether there is a specific interaction between dynein and Ndc80. Using low nanomolar concentrations (1 nM) of both the proteins, we found that approximately 40% of Ndc80 colocalize with dynein on microtubules (Fig. 6*L*). On the other hand, at higher concentrations (50 nM for both), we observed a mix of complete codecoration and mutually exclusive microtubule binding for both the proteins (Fig. S6*D*). Together, these results show that Ndc80 and dynein co-operate together to regulate each other’s microtubule binding activity in order to restrict the formation of too many initial lateral kMT attachments, while favoring plus-end capture events. The capture and subsequent poleward motion of kinetochores also appear to be regulated by the phosphorylation status of kinetochore Ndc80. Since Ndc80 at prometaphase kinetochores is phosphorylated by Aurora B, this can facilitate the conditions that favor dynein-driven poleward motion of kinetochores. It is also interesting to note that this could be an efficient mechanism *in vivo* to regulate the necessity of end-on attached, monoriented kinetochores to be transported toward the spindle pole based on their position relative to the spindle and the phosphorylation status of Ndc80.

### Additional considerations and conclusions

Overall, our study sheds light on the mechanism of a previously reported but critically understudied function of the human Ndc80 complex during early mitosis. The Ndc80 complex localizes to kinetochores during mitosis from prophase to anaphase (19, 31, 32). The main function of Ndc80 in stabilizing load-bearing kMT attachments in metaphase has been well studied. However, the evidence for Ndc80’s role in kMT capture is primarily derived from studies on chromosome congression rather than from an in-depth analysis of the actual attachment process. Its localization to kinetochores in early mitosis and strong affinity for binding to microtubules thus led us to first test for the requirement of Ndc80 for kinetochore capture during early mitosis. It has been previously shown that chromosomes captured by spindle microtubules move poleward rapidly, *via* lateral kMT attachments and motility mediated by dynein (15, 29, 49). *In vitro* analysis showed that microtubule binding to kinetochores is reduced by dynein/dynactin inhibition (29) and that kinetochore dynein prevents premature stabilization of load-bearing, end-on kMT attachments (37, 45, 79, 80). Our data suggest that dynein, however, is not critically required for a large fraction of initial kMT attachments that are formed during early mitosis in human cells (Fig. S2, *C* and *D*). Further, our studies suggest that other kinetochore proteins, including dynein-dynactin, CLIP-170, Spindly, and CENP-E, which localize to prometaphase kinetochores and are important for chromosome alignment also do not possess a significant role in the initial kMT attachment process during early mitotic progression in normal cells (Fig. S2*C*). However, in the scenario where normal mitotic progression is severely delayed by defective kMT capture in Ndc80-inhibited cells, we predict that the abovementioned microtubule-binding factors are likely to compensate for the Ndc80 loss of function and rescue chromosome alignment to a considerable extend.

**Figure 7:**
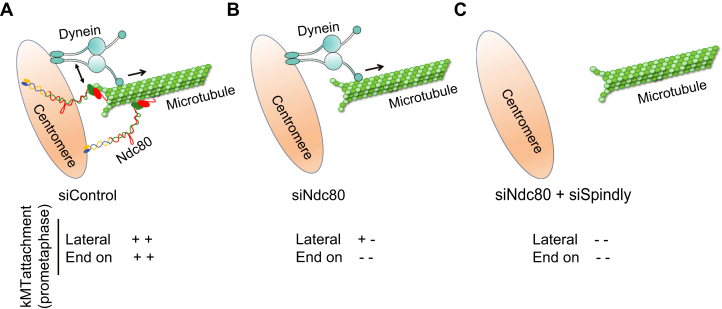
**A cartoon depicting the coordinated action of Ndc80 and dynein in the formation of initial kMT attachments during early mitosis.** During the kMT capture process, Ndc80 at the kinetochore is involved in attaching to the microtubules, not only *via* dynamic lateral kMT contacts but also *via* coordination with dynein to form dynamic end-on contacts (*A*). The kinetochore motors, dynein (*A*, *B*) and CENP-E (not depicted), assist Ndc80 by making lateral kMT contacts and driving kinetochore motility. In the absence of the Ndc80 complex, only about ∼30% of lateral kMT contacts are made, mediated by dynein and/or CENP-E (*B*). Once the initial attachment is accomplished, all kinetochores depend on dynein for spindle poleward motility, an event that is also needs to be coordinated with the Ndc80 complex to constitute a productive kMT capture event (*A*). Loss of function of both the Ndc80 complex as well as dynein leads to the vast majority of kinetochores being not captured (*C*) and contributing to the observed kinetochore null phenotype. CENP-E, centrosome-associated protein E; kMT, kinetochore-microtubule; Ndc80, nuclear division cycle 80.

More importantly, in this study, we show that the kinetochore-bound Ndc80 complex plays an essential role in initial kMT attachment formation during early mitosis in humans (Fig. 7, *A*–*C*). Original studies in yeast had found that the Ndc80 complex is important to from lateral kMT interactions during closed mitosis (13). While it is not clear if such a scenario definitely exists during open mitosis in human cells, initial studies on the Ndc80 complex’s role in chromosome congression along with the *in vitro* studies analyzing microtubule binding of the Ndc80 complex suggest that a considerable faction of these attachments that are facilitated by Ndc80 occur laterally to the microtubule sidewalls. These attachments are similar to those made by the microtubule motors, which are also lateral in nature, even though our results indicate that these factors only account for a smaller fraction of lateral capture events as compared to Ndc80. The significance of our work is that we have identified a novel form of coordination between Ndc80 and the dynein motor that favors attachments that are possibly dynamic end-on contacts between microtubule plus-ends and mono-oriented kinetochores (Fig. 7*A*). Our results suggest that such Ndc80-dependent end-on attachments are also critical for chromosome capture, the loss of which, leads to severe defects in kMT capture (the kinetochore-null phenotype) observed after the combined inhibition of the Ndc80 complex and dynein (Fig. 7, *A*–*C*).

In addition to the abovementioned novel form of coordination that facilitates plus-end capture, the lateral attachments mediated by the Ndc80 complex also possibly requires another mode of coordination with the dynein motor where the microtubules captured by the Ndc80 is passed on to dynein to drive the poleward motility of kinetochores. Our results suggest that, in this ‘hand-over’ mechanism, a major fraction of the initial kMT attachments are still mediated by the Ndc80 complex which in turn hands-over the captured kinetochore to dynein for poleward kinetochore motility (Fig. 7*A*). This nonprocessive but moderately robust kMT interactions in early mitosis facilitated by the Ndc80 complex would be critical to enhance the efficiency of kMT capture as prometaphase cells wait for the build-up of kinetochore dynein levels so that they can switch to dynamic kMT interactions and processive kinetochore motility. It is interesting to note that the Aurora B phosphorylated version of the Ndc80 complex still retains considerable affinity for microtubules to favor initial kMT contacts during early mitosis (Fig. S5*C*) (78) (this study). Once the chromosomes attain proper alignment and biorientation, the Ndc80 complex at kinetochores serves its well-established function in the formation of load-bearing kMT attachments during metaphase and anaphase.

As expected, the role of the Ndc80 complex in initial kMT attachments is dependent on its key microtubule-binding elements, the CH domain and the tail domain, at the N terminus of the Hec1 subunit. The phosphorylation of these domains by Aurora B, possibly coupled with kinetochore phosphatase activity maintains a reduced affinity state of the Ndc80 complex for microtubules in prophase and prometaphase (18, 81). Our studies suggest that this version of the Ndc80 complex with the reduced affinity for microtubules is essential for initial kMT attachments during early mitosis. In support of this notion, the nonphosphorylatable Hec1^9A^ mutant showed efficient microtubule binding to facilitate proper kMT capture similar to what is observed in cells rescued with Hec1^WT^. On the other hand, the Hec1^K166D^ mutant does not allow the formation of proper initial kMT attachments due to a conformational change in Hec1 that inhibits the CH domain binding to microtubules. Thus, as for load-bearing kMT attachments in metaphase, the initial kMT attachments are also regulated by Aurora B-mediated phosphoregulation of the N-terminal tail domain and the CH domain of the Hec1 subunit.

As mentioned earlier, it is worth noting that the Ndc80 complex could be instrumental in facilitating initial kMT attachments by two different mechanisms, both dependent on its ability to bind microtubules. The first mechanism strongly supported by our observations is that the microtubule-binding activity of the complex is required to directly bind to *de novo* microtubules emerging from the spindle poles *via* the formation of lateral and/or end-on dynamic kMT contacts. The second equally plausible mechanism, the observation of which is limited by the resolution of our imaging technology is that the microtubule-binding activity of the Ndc80 complex could be essential for the formation of *de novo* microtubules originating at the kinetochores. Acentrosomal microtubules that are generated by such an Ndc80-mediated mechanism could it turn couple with the Augmin complex-dependent microtubule branching/bundling within the mitotic spindle (82, 83), thus contributing to kinetochore capture and kMT maturation during early mitosis.

Apart from the mechanisms of coordination that have already been discussed, the additive nature of the Ndc80 and dynein co-inhibition phenotype for the formation of initial kMT attachment suggest additional, possibly indirect, means of coordination between these two kinetochore complexes. One intriguing possibility is that the Ndc80 complex might be important to relieve the autoinhibition of the dynein motor that in turn could ‘prime’ the motor for processive minus-end directed motility. It has been proposed that the dynein motor is maintained in an autoinhibited state prior to cargo binding (84, 85). Another possibility is that the interaction contributes to the kinetochore binding of at least a small fraction of dynein molecules. However, the severe defects in kinetochore capture observed after inhibiting Ndc80 as compared to the relatively minor phenotype obtained after dynein inhibition suggests that the possible loss of kinetochore dynein is likely not the main cause for defective capture after Ndc80 inhibition. Recent studies have also reported that the premature stabilization of Ndc80-mediated load-bearing kMT attachments during early mitosis is inhibited by kinetochore dynein (37, 45, 80). From our current findings, we surmise that the function of Ndc80 complex in forming initial kMT attachments during early mitosis should precede the onset of inhibition of its function in kMT attachment stabilization by dynein. Many possibilities aside, it is currently not clear how the interaction we observe between dynein and the Ndc80 complex in prometaphase extracts contribute to the coordinated function between these complexes for efficient kinetochore capture. Further studies are clearly required to delineate the molecular underpinnings of dynein-Ndc80 coordination during early mitosis.

## Experimental procedures

### Cell culture, transfections, and drug treatments

HeLa and RPE1 cells were grown in Dulbecco's modified Eagle medium (DMEM, Life Technologies) supplemented with 10% fetal bovine serum at 37 ^°^C in humidified atmosphere with 5% CO_2_. The doxycycline (dox)-inducible CRISPR/Cas9 Nuf2 knockout HeLa cell line (generous gift from Iain Cheeseman, MIT) was maintained in the absence of dox.

For RNAi experiments, cells were transfected at 30 to 50% confluence using Dharmafect 2 (Dharmacon) according to the manufacturer’s instructions and analyzed 48 to 72 h after transfection. For rescue experiments, RNAi-refractory constructs were transfected into cells using Lipofectamine 3000 (Life Technologies) for 12 h followed by siRNA transfection. Cells were prepared for analysis after 48 h of siRNA transfection.

For testing kinetochore microtubule capture, cells were treated with STLC (5 μM) for 2 h prior to fixation to arrest them in early mitosis with monopolar mitotic spindles. Nocodazole (3 μM) was used to depolymerize the microtubule. To inhibit Aurora B kinase, 5 μM ZM (APExBio) was added to the medium, and cells were fixed after 1 h of incubation. Cells were treated with 10 μM MG132 to prevent mitotic exit after the inhibition of Aurora B kinase. Cas9 expression was induced by 1 μM doxycycline hyclate (Sigma).

siRNAs targeting 5′ UTR Ndc80 (86), 3′ UTR Dynein (45), and siRNAs targeting cDNAs for CLIP-170 and CENP-E (44), Nuf2 (25), and Knl1 (46) were used this study. The Smart Pool ON-TARGET plus siRNAs was used for Spindly knockdown (45). All siRNAs were used at 100 nM concentration except for dynein and CLIP-170 (200 nM).

### Antibodies

The primary monoclonal mouse antibodies used were anti-Hec1 9G3 (Abcam, ab3613) for immunoflourescence (IF) at 1:400 and for WB at 1:1000; anti-dynein intermediate chain (DIC) clone 74.1 (EMD Millipore, MAB1618) for IF at 1:300 and for WB at 1:1000, anti-CENP-E 1H12 (Abcam, ab5093) IF at 1:400, and anti-α-tubulin DM1A (Santa Cruz, Sc32293) IF at 1:750 and WB at 1:1000. The primary rabbit polyclonal antibodies used were anti-Hec1 (Bethyl Laboratories, A300-771A), anti-CLIP-170 (Santa Cruz, H-300) for IF at 1:750, anti-Zwint1 (Bethyl, A300-781A) for IF at 1:400, anti-Spindly (a gift from Dr Reto Gassmann, UC San Diego) for IF at 1:5000 and for WB at 1:1000; and anti-CENP-E (Boster, M04553-1) for WB at 1:1000. We also used a human anti-ACA antibody (Immunovision, HCT-0100) at 1:500 for IF.

### Immunofluorescence

HeLa cells grown on a glass coverslip were fixed in cold methanol (−20 ^°^C) or 4% formaldehyde after pre-extraction with 0.1% Triton X-100 followed by blocking with 3% bovine serum albumin in PBS and incubated with primary antibodies for 1 h at 37 ^°^C, followed by washing with PBS (137 mM NaCl, 2.7 mM KCl, 10 mM Na_2_HPO_4_ and 1.8 mM KH_2_PO_4_, pH 7.4) supplemented with 0.02% Triton X-100. The secondary antibodies coupled with Alexa-Fluor-488/647 or Rhodamine Red-X (Jackson ImmunoResearch Laboratories, Inc) were used at a dilution of 1:250, and DNA was counterstained with 1 mg/ml DAPI.

For nocodazole washout assay, cells were treated with nocodazole (3 μM) for 3 h and transferred to DMEM medium at 37 ^°^C after washing out nocodazole three times with PBS and once with warm DMEM medium. Cells were fixed at 0- and 10-min incubation with DMEM medium.

### Image acquisition and analysis

For image acquisition, three-dimensional stacks were obtained through the cell using a Nikon Eclipse TiE inverted microscope equipped with a Yokogawa CSU-X1 spinning disc, an Andor iXon Ultra888 EMCCD camera, and an ×60 or ×100 1.4 NA Plan-Apochromatic DIC oil immersion objective (Nikon). For fixed cell experiments, images were acquired at room temperature as Z-stacks at 0.2 μm intervals controlled by NIS-elements software (Nikon). Images were processed in Fiji ImageJ and Adobe Photoshop CC 2018 and represent maximum-intensity projections of the required z-stacks.

For live-cell imaging, HeLa cells stably expressing both mCherry H2B and GFP-α-tubulin or only GFP-H2B were cultured in 35 mm glass-bottomed dishes (MatTek Corporation). Before 30 min of imaging, cell culture medium was changed to prewarmed L-15 medium (Gibco) supplemented with 20% fetal bovine serum and 20 mM Hepes, pH 7.0. Live experiments were carried-out in an incubation chamber for microscopes (Tokai Hit Co, Ltd) at 37 ^°^C and 5% CO_2_. Image recording was initiated immediately after adding MG132 (unless otherwise stated) using an ×60 1.4 NA Plan-Apochromatic DIC oil immersion objective mounted on an inverted microscope (Nikon) equipped with an Andor iXon Ultra888 EMCCD camera or an Andor Zyla 4.2 plus sCMOS camera. Twelve 1.2 μm-separated z-planes covering the entire volume of the cell were collected at every 10 min up to 12 h.

To quantify the kinetochore motions, images were captured at 0.8 μm intervals every 5 s. Sister kinetochore pairs chosen for analysis were located at the periphery of the monopolar spindle. Kinetochore movements were tracked using GFP-CENP-A fluorescence using the manual-tracking tool in ImageJ software.

To measure the distance between kinetochore and nearest microtubule loci (attached or unattached), we manually selected a suitable image (captured at 0.2 μm intervals) from a z stack carrying the concerned kinetochore and microtubule. We then calculated the distance of separation between the perceived center of the kinetochore and the end of the microtubule using ImageJ software.

All images and videos were processed in ImageJ/Fiji ImageJ and Adobe Photoshop CC 2017. All images represent maximum-intensity projections of the required z-stacks. Statistical tests performed are specified in figure legends.

### Protein purification

Ndc80 proteins used were a kind gift from the Deluca and Grishchuk laboratories. Plasmid for expression and purification of the Human dynein complex was purchased from Addgene (Cat #111903) Expression and purification of this protein done following published protocol (77) with a few modifications as detailed in Chakraborty *et al.*, Biorxiv 2020 (87). Untagged tubulin protein was purchased from PurSolutions (Cat # 032005), and X-Rhodamine-labeled tubulin protein was a kind gift from Ted Salmon’s laboratory.

### Western blotting

Cell lysates were prepared with lysis buffer [150 mM KCl, 75 mM Hepes of pH 7.5, 1.5 mM EGTA, 1.5 mM MgCl_2_, 10% glycerol, 0.1% NP-40, 30 mg/ml DNase, 30 mg/ml RNase, complete protease inhibitor cocktail (Roche), and complete phosphatase inhibitor cocktail (Sigma)]. Protein concentration of cell lysate was measured using the Coomassie protein assay kit (Thermo Scientific). Proteins were separated on SDS-PAGE, electroblotted onto a nitrocellulose blotting membrane (Amersham, GE Healthcare), and subjected to immunodetection using appropriate primary antibodies. Blocking and antibody incubations were performed in 5% nonfat dry milk. Proteins were visualized using horseradish peroxidase-conjugated secondary antibodies diluted at 1:2000 (Amersham) and the ECL system, according to the manufacturer’s instructions (Thermo Scientific).

### Total internal reflection fluorescence microscopy

#### Microtubule assembly

Taxol stabilized microtubules were prepared from mixing on ice the following; 4 μl 10 mg/ml unlabeled tubulin, 0.4 μl 5 mg/ml X-rhodamine-labeled tubulin, 20% glycerol, and 1 mM Mg-GTP in MRB80 (80 mM Pipes, 4 mM MgCl_2_, 1 mM EGTA, pH 6.9). The mixture was then immediately placed into 37 ^°^C water bath for 30 min. After polymerization, 10 μM Taxol in MRB80 was added to the polymerized mixture. The mixture was then further incubated in 37 ^°^C water bath for additional 10 min followed by addition of 50 μl 10 μM Taxol in MRB80 (buffer B). The polymerized mix was then centrifuged at 12,000 *×g* for 10 min at RT. The supernatant was discarded, and the resulting microtubule pellet was resuspended in 50 μl buffer B and was kept in dark for immediate use. Rhodamine-labeled tubulin was obtained from Dr Ted Salmon's laboratory at UNC-Chapel Hill and rhodamine–biotin–labeled tubulin was prepared in an analogous way.

#### Microtubule capture assay

A microscopy perfusion chamber was prepared by attaching an acid washed coverslip (22 × 22 mm, thickness 1 1/2, Corning) over a precleaned glass slide (75 × 25 mm, thickness 1 mm, Corning) using a double-sided tape (Scotch) resulting in a chamber volume of ∼10 μl. For more details, please see TIRF section in (88). Acid washed coverslips were further cleaned by holding under flame for a couple of seconds prior to use. Solutions were then exchanged into the perfusion chamber by micropipette and filter paper. A solution of indicated proteins or mixture of proteins (at concentrations 100 nM each) in MRB80 were flown into the chamber and incubated for 30 min inside a moistened slide holder to prevent drying. The chamber was then washed with image buffer (MRB80 supplemented with 10 μM Taxol, 0.6 mg/ml *κ*-casein, 4 mM DTT, 50 mM glucose (#G8270, Sigma), 0.2 mg/ml, catalase (#C9322, Sigma), and 0.4 mg/ml glucose oxidase (#G7141, Sigma) followed by incubation for 5 min. A solution of Taxol-stabilized MTs in image buffer was flown into the chamber and incubated for 10 min. Unbound MTs were then washed out by flowing in image buffer. The chamber was sealed and then images were collected using microscopy setting below.

For the titration experiment, dynein was labeled with alexa488 following established protocol (77) and full-length Ndc80 protein [purification described elsewhere (89)] was labeled with Alexa647 dye using NHS-Alexa 647 as described before (90). The proteins were immobilized as explained before using 100 nM dynein and varying concentrations of Ndc80. Microtubules labeled with rhodamine (ex. 560 nm) dye were used in this assay, and gliding motion of the microtubule was recorded for 5 min. Brightness was quantified by imaging in appropriate imaging channel for dynein (ex. 488 nm) and Ndc80 (ex. 640 nm) and following the recommended microscopy setting below. Mean intensity of each pixel is then quantified using Fiji, and gliding velocities for each condition were quantified as above. For single-molecule colocalization assay, we used nonfluorescent Taxol stabilized-biotin labeled microtubule, and microtubules were immobilized on the surface as described elsewhere (90). A solution of premixed dynein (1 nM) and Ndc80 (1 nM) solution in image buffer was flown into the chamber following visualization in appropriate channel.

Nikon Ti-E inverted microscope equipped with Andor iXon3 CCD camera (Cambridge Scientific), 1.49 × NA, and 100 × oil objective. The microscope produced a 512 × 512-pixel images with 0.16 μm per pixel resolution in both *x* and *y* directions. Images of the Ndc80-GFP and microtubules were collected using 488 nm and 560 nm laser excitation, respectively. NIS-elements software (Nikon) was used for data acquisition with 100 ms exposure times. Images were further processed using FIJI. For counting number of microtubules in a field, the whole field of view (512 × 512) was chosen over several frames and multiple independent trials. Gliding velocity were calculated from kymographs made with FIJI.

### Statistical analysis

The statistical analyses for scattered graphs were carried out using GraphPad Prism software (version 8.1.0). Samples for analysis in each dataset were acquired in the same experiment, and all samples were calculated at the same time for each dataset. A two-sided *t* test was used for comparison of average for bar graphs.

## Data availability

All data have been provided within the manuscript or the supporting information.

## Supporting information

This article contains supporting information.

## Conflict of interest

The authors declare no conflict of interest with the contents of this article.

## Supporting information

Movie S1 Video of the dynamics of kinetochore pairs in HeLa cells stable expressing GFP-CENPA and GFP-Tubulin and treated with control siRNA. 0.6 μm z sections were acquired at 15 sec intervals. Bars, 5 μm. Video speed is 5 frames per second.

Movie S2 Video of the dynamics of kinetochore pairs in HeLa cells stable expressing GFP-CENPA and GFP-Tubulin and treated with siRNA for Ndc80. 0.6 um z sections were acquired at 15 sec intervals. Bars, 5 μm. Video speed is 5 frames per second.

Movie S3 Video of kinetochore capture by microtubules during early prometaphase stage in HeLa cells stably expressing H2B-mCherry, GFP-α-tubulin, GFP-CENP-A and treated with control siRNA. Images were captured starting from the nuclear envelope breakdown until the time when chromosome alignment was attained. 0.6 μm z sections were acquired at 1 min intervals. Bars, 5 μm. Video speed is 5 frames per second.

Movie S4 Video of kinetochore capture by microtubules during early prometaphase stage of HeLa cells stably expressing H2B-mCherry, GFP-α-tubulin, GFP-CENP-A and treated with siRNA for Ndc80. Images were captured starting from the nuclear envelope breakdown until the time when complete chromosome alignment was expected to be attained (for around 30 min). 0.6 μm z sections were acquired at 1 min intervals. Bars, 5 μm. Video speed is 5 frames per second.

Movie S5 Video of chromosome congression during early prometaphase (where initial kMT attachment occurs) in parental HeLa cells treated with Hoechst to visualize the chromosomes. Images were captured starting from the nuclear envelope breakdown until the time when complete chromosome alignment was attained. 1 μm z sections were acquired at 2 min intervals. Bars, 5 μm. Video speed is 5 frames per second.

Movie S6 Video of chromosome congression during early prometaphase where initial kMT attachment occurs of CRISPR/Cas9 Nuf2^KO^ HeLa cells treated with Hoechst to visualize the chromosomes. Images were captured starting from the nuclear envelope breakdown until the time when complete chromosome alignment was expected to be attained (for around 30 min). 1 um z sections were acquired at 2 min intervals. Bars, 5 μm. Video speed is 5 frames per second.

Movie S7 Video showing the behavior of kinetochores in HeLa cells expressing H2B-mCherry, GFP-α-tubulin and treated with control siRNA. Imaging was started after 30 min of adding MG132 and ZM. For the particular mitotic cell shown, images were captured starting from the nuclear envelope breakdown until the time when chromosomes were expected to align at the metaphase plate (for around 30 min). 0.6 μm z sections were acquired at 2 min intervals. Bars, 5 μm. Video speed is 5 frames per second.

Movie S8 Video demonstrating the kinetochore-null phenotype of HeLa cells expressing H2B-mCherry, GFP-α-tubulin and treated with siRNA for Ndc80. Imaging was started after 30 min of adding MG132 and ZM. For the particular mitotic cell shown, images were captured starting from the nuclear envelope breakdown until the time when chromosomes were expected to align at the metaphase plate (for around 30 min). 0.6 μm z sections were acquired at 2 min intervals. Bars, 5 μm. Video speed is 5 frames per second.

Movie S9 Taxol-stabilized microtubules gliding over immobilized dynein. Video showing gliding motion of Taxol-stabilized Rhodamine microtubules visualized with 560 nm excitation laser in TIR-FM. Video is played 15 times faster rate than real. Scale bar 5 μm.

Movie S10 Taxol-stabilized microtubules gliding over immobilized dynein plus Ndc80^WT^ bonsai. Video showing gliding motion of Taxol-stabilized Rhodamine microtubules visualized with 560 nm excitation laser in TIR-FM. Video is played 15 times faster rate than real. Scale bar 5 μm.

Movie S11 Taxol-stabilized microtubules gliding over immobilized dynein plus Ndc80^9D^ bonsai. Video showing gliding motion of Taxol-stabilized Rhodamine microtubules visualized with 560 nm excitation laser in TIRF. Video is played 15 times faster rate than real. Scale bar 5 μm.

Movie S12 Lateral landing of Taxol-stabilized microtubule over a lawn of dynein plus Ndc80^9D^ bonsai. Video showing initial attachment (landing) of Taxol-stabilized Rhodamine microtubules on the surface coated with Dynein and Ndc80^9D^ bonsai in a gliding assay. Video is played 15 times faster rate than real. Scale bar 5 μm.

Movie S13 End on landing of Taxol stabilized microtubule over a lawn of dynein plus Ndc80^9D^ bonsai. Video showing initial attachment (landing) of Taxol-stabilized Rhodamine microtubules on the surface coated with Dynein and Ndc80^9D^ bonsai in a gliding assay. Note the microtubule initiates contacts with its end which was later identified to be plus end based on the direction of motion of the microtubule. Video is played 15 times faster rate than real. Scale bar 5 μm.

Supporting Figure S1 **Immunofluorescence staining showing the loss of kinetochore Ndc80 and perturbed initial kMT attachments after different modes of Ndc80 inhibition.***A*, Quantification of intensity of dynein in Figure 1*A*. A total of 50 kinetochores were analyzed from at least five different prophase and prometaphase cells. *B*, Immunofluorescence staining of mitotic HeLa cells depleted of Ndc80 (*top two panels*) or knocked out of Nuf2 (*middle two panels*) or of RPE1 cells depleted of Nuf2 (*bottom two panels*) respectively in comparison to control or parental cells. The cells were stained for Ndc80 (*green*), a kinetochore marker CREST (*red*) with the chromosomes counterstained using DAPI. Bars, 5 μm. *C*, Quantification of unattached, monotelic (one kinetochore attached) and syntelic (both kinetochores attached) of cells from Figure 1*D*. Error bars represent S.D. from three independent experiments. For each experiment, on average ∼20 kinetochore pairs from ten different monopolar cells were examined. ∗∗∗∗*P* < 0.0001 (Student’s *t* test). *D*, Immunofluorescence staining of STLC-treated mitotic HeLa cells knocked out of Nuf2 (*bottom panel*) in comparison to parental cells (*top panel*) and stained for α-tubulin (*green*), a kinetochore marker Zwint1 (*red*) with the chromosomes counterstained using DAPI. Bars, 5 μm. Inset shows the kMT attachment status of individual kinetochore pairsafter Nuf2 KO. Bar (applies to all insets), 1 μm. *E*, Quantification of the status of kMT attachments in cells from *B*. Error bars represent S.D. from three independent experiments. For each experiment, ten monopolar cells were examined. ∗∗∗∗*P* < 0.0001 (Student’s *t* test).

Supporting Figure S2 **Localization of different kinetochore proteins in early prometaphase and analysis of their role in initial kMT capture.***A*, Immunofluorescence staining of early prometaphase HeLa cells with different kinetochore proteins as indicated (*green*), and a kinetochore marker CREST (*red*) with the chromosomes counterstained using DAPI. Bars, 5 μm. *B*, Western blot analysis of HeLa cells treated with indicated siRNAs. α-tubulin was used as a loading control. *C*, Immunofluorescence staining of STLC-treated mitotic prometaphase cells depleted of the indicated target proteins as compared to control HeLa cells, and stained for α-tubulin (*green*), a kinetochore marker Zwint1 or CENP-A (*red*) with the chromosomes counterstained using DAPI. Bars, 5 μm. Inset shows the kMT attachment status of individual kinetochore pairs in the conditions indicated. Bar (applies to all insets), 1 μm. *D*, Quantification of the status of kMT attachments in cells from *C*. Error bars represent S.D. from three independent experiments. For each experiment, on an average ∼20 kinetochore pairs from ten monopolar cells were examined. ∗∗∗∗*P* < 0.0001 (Student’s *t* test).

Supporting Figure S3 **Nocodazole wash out assay to assess kinetochore capture by de novo microtubules early in mitosis after different modes of Ndc80 inhibition.***A*, Cells were subjected to the indicated perturbation, treated with the indicated drug, and fixed according to the scheme. *B* and *D*, Immunofluorescence staining of mitotic HeLa cells knocked out of Nuf2 (*B*) or of mitotic RPE1 cells depleted of Nuf2 (*D*) in comparison to control or parental cells respectively and immunostained for α-tubulin (*green*), a kinetochore marker Zwint1 (*red*) with the chromosomes counterstained using DAPI. Bars, 5 μm. Inset shows the kMT attachment status of individual kinetochore pairs in the indicated conditions. Bar (applies to all insets), 1 μm. *C* and *E*, Quantification of the status of kMT attachments in cells from *B* and *D*. Error bars represent S.D. from three independent experiments. For each experiment, ten mitotic cells were examined. ∗∗∗∗*P* < 0.0001 (Student’s *t* test).

Supporting Figure S4 **Further characterization of the kinetochore null phenotype and dynein kinetochore localization in Ndc80-inhibited cells.***A* and *B*, Mitotic HeLa cells depleted of Ndc80 (*A, bottom panel*) or knocked out of Nuf2 (*B*, *bottom panel*) as compared to control^RNAi^ (*A*, *top panel*) or parental control cells (*B*, *bottom panel*), and followed by the indicated drug treatments, were immunostained for α-tubulin (*green*), a kinetochore marker Zwint1 or CENPA (*red*) with the chromosomes counterstained using DAPI. Bars, 5 μm. *C*, Quantification of the frequency of mitotic cells with null kinetochores (see main text for more details) in samples from *A* and *B*. Error bars represent S.D. from three independent experiments. For each experiment, 200 mitotic cells were examined. ∗∗∗∗*P* < 0.0001 (Student’s *t* test).

Supporting Figure S5 **Ndc80**^**9D**^**mutant captures/binds microtubules in both the TIR-FM geometries tested.***A*, An SDS-PAGE electrophoretic gel of purified human whole cytoplasmic dynein complex used in the study. *B*, Images showing binding of Ndc80^9D^ bonsai-GFP to Taxol-stabilized Rhodamine-labeled microtubules immobilized on coverslips. Scale bar, 5 μm. *C*, Capture of microtubules by Ndc80^wt^ or Ndc80^9D^-bonsai in the geometry used for the microtubule-landing experiments as in Figure 6 where these proteins were immobilized on to coverslips and Rhodamine microtubules were floated-in. Scale bar, 5 μm. *D*, Plot of mean surface intensity of GFP-tagged Ndc80 proteins. The ‘dynein only’ measurements indicate the background. N = 3 independent trials.

Supporting Figure S6 **Characterization of Dynein Ndc80 interaction using multiple parameters.***A*, Plot of mean surface density of dynein^alexa488^(*left*), and Ndc80^alexa647^(*right*) as a function of surface Ndc80^alexa647^ concentration. Data is Mean ± SEM. *B*, Fraction of the various kinds of microtubules landing behavior with dynein plus Ndc80 (consolidated numbers for Ndc80 ^9D and wt^-bonsai, *B* and *C* from above) immobilized on the surface. t1, t2, and t3 indicate different trials on different days. *C*, Quantification of the number of microtubules bound to the surface for indicated proteins. Bars, mean ± SD. *D*, Montage showing Dynein^alexa488^ (*top*, concentration 50 nM), Ndc80^alexa647^ (*center*, concentration 50 nM) and merge (*bottom*) bound to taxol stabilized microtubule (not imaged). Bar is 5 μm.
