# Supplementary figures and images for: Coordination between the Ndc80 complex and dynein is essential for microtubule plus-end capture by kinetochores during early mitosis
Source: J Biol Chem. 2023 Apr 14;299(6):104711. doi: 10.1016/j.jbc.2023.104711 (PMC10206188; doi:10.1016/j.jbc.2023.104711)

Figure S1

A

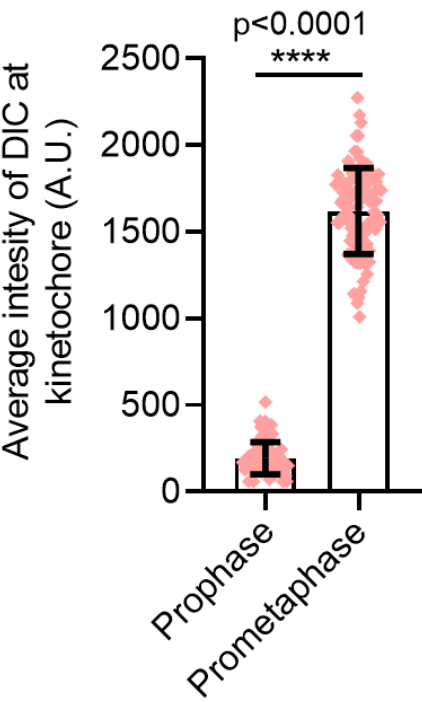

B

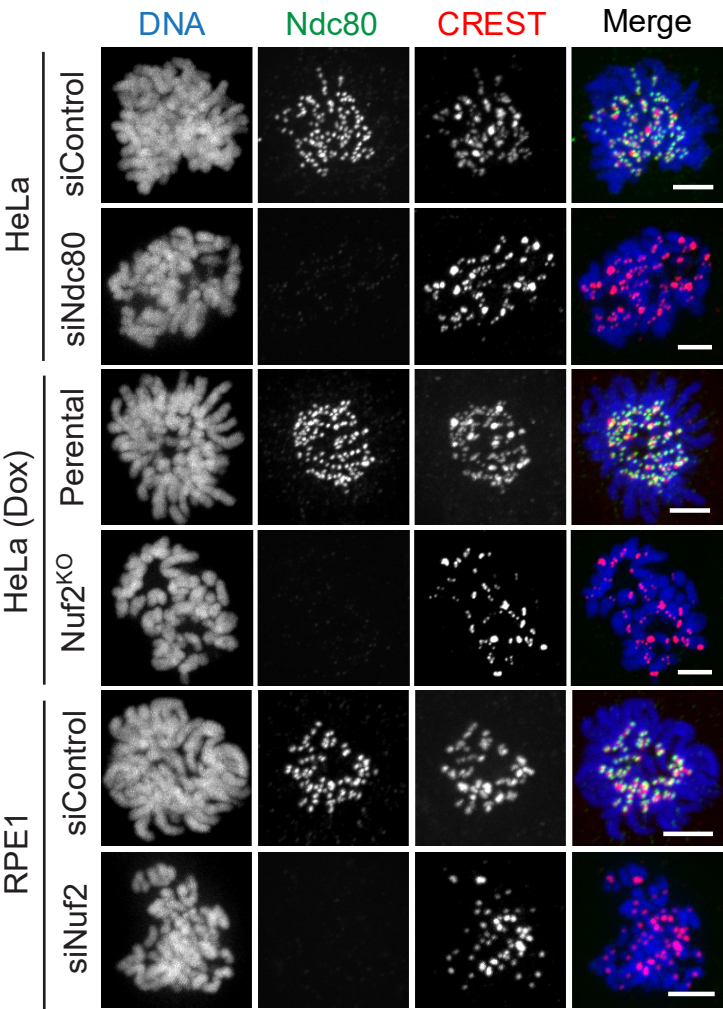

C

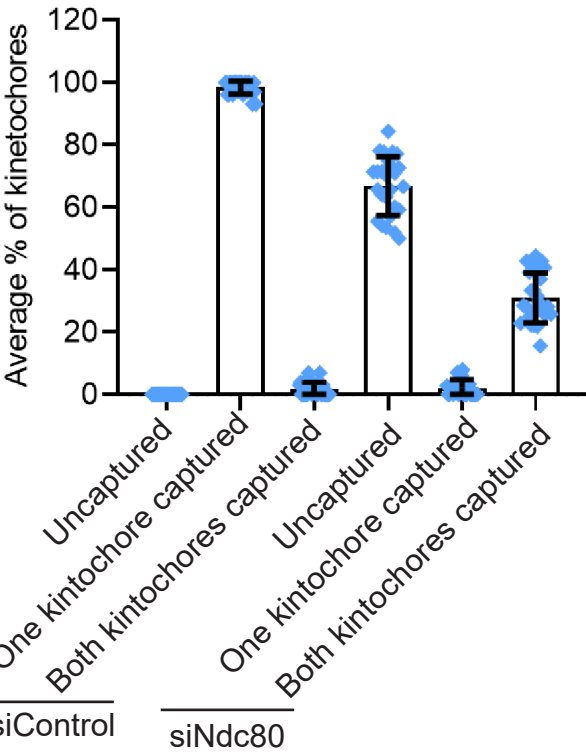

D

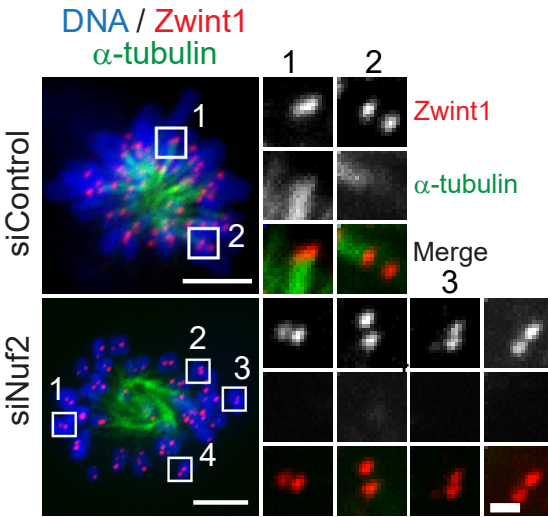

E

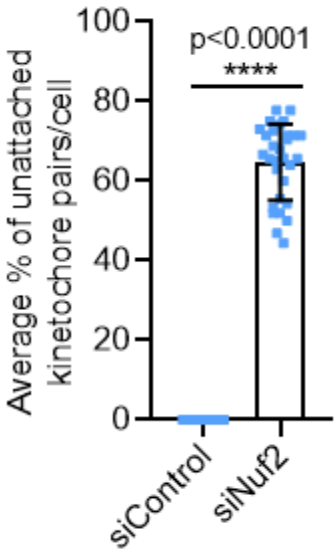

Supplement: Supporting Figure S1 — Immunofluorescence staining showing the loss of kinetochore Ndc80 and perturbed initial kMT attachments after different modes of Ndc80 inhibition.A, Quantification of intensity of dynein in Figure 1A. A total of 50 kinetochores were analyzed from at least five different prophase and prometaphase cells. B, Immunofluorescence staining of mitotic HeLa cells depleted of Ndc80 (top two panels) or knocked out of Nuf2 (middle two panels) or of RPE1 cells depleted of Nuf2 (bottom two panels) respectively in comparison to control or parental cells. The cells were stained for Ndc80 (green), a kinetochore marker CREST (red) with the chromosomes counterstained using DAPI. Bars, 5 μm. C, Quantification of unattached, monotelic (one kinetochore attached) and syntelic (both kinetochores attached) of cells from Figure 1D. Error bars represent S.D. from three independent experiments. For each experiment, on average ∼20 kinetochore pairs from ten different monopolar cells were examined. ∗∗∗∗P < 0.0001 (Student’s t test). D, Immunofluorescence staining of STLC-treated mitotic HeLa cells knocked out of Nuf2 (bottom panel) in comparison to parental cells (top panel) and stained for α-tubulin (green), a kinetochore marker Zwint1 (red) with the chromosomes counterstained using DAPI. Bars, 5 μm. Inset shows the kMT attachment status of individual kinetochore pairsafter Nuf2 KO. Bar (applies to all insets), 1 μm. E, Quantification of the status of kMT attachments in cells from B. Error bars represent S.D. from three independent experiments. For each experiment, ten monopolar cells were examined. ∗∗∗∗P < 0.0001 (Student’s t test). [file mmc14.pdf]

Figure S2

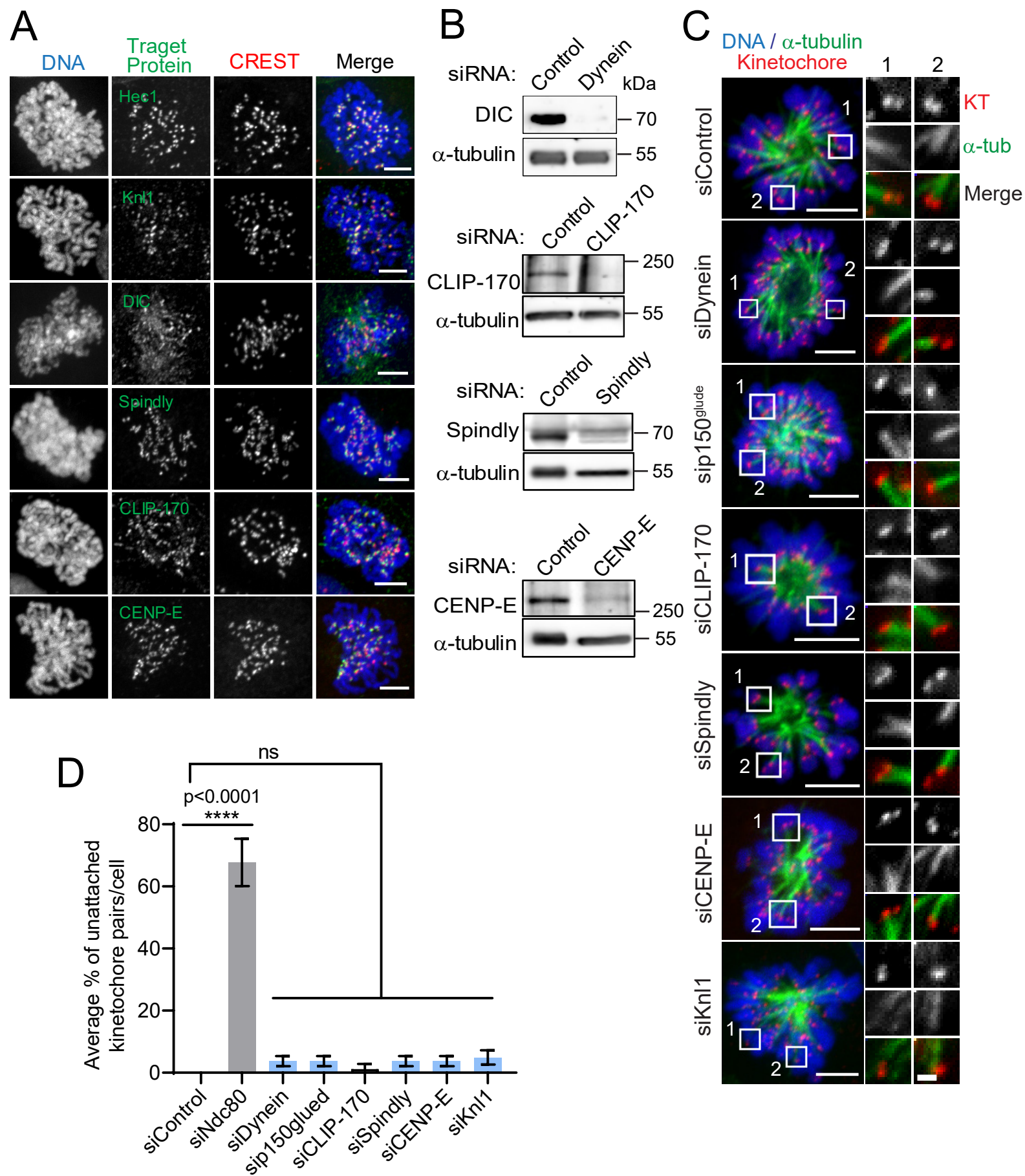

Supplement: Supporting Figure S2 — Localization of different kinetochore proteins in early prometaphase and analysis of their role in initial kMT capture.A, Immunofluorescence staining of early prometaphase HeLa cells with different kinetochore proteins as indicated (green), and a kinetochore marker CREST (red) with the chromosomes counterstained using DAPI. Bars, 5 μm. B, Western blot analysis of HeLa cells treated with indicated siRNAs. α-tubulin was used as a loading control. C, Immunofluorescence staining of STLC-treated mitotic prometaphase cells depleted of the indicated target proteins as compared to control HeLa cells, and stained for α-tubulin (green), a kinetochore marker Zwint1 or CENP-A (red) with the chromosomes counterstained using DAPI. Bars, 5 μm. Inset shows the kMT attachment status of individual kinetochore pairs in the conditions indicated. Bar (applies to all insets), 1 μm. D, Quantification of the status of kMT attachments in cells from C. Error bars represent S.D. from three independent experiments. For each experiment, on an average ∼20 kinetochore pairs from ten monopolar cells were examined. ∗∗∗∗P < 0.0001 (Student’s t test). [file mmc15.pdf]

# Figure S3

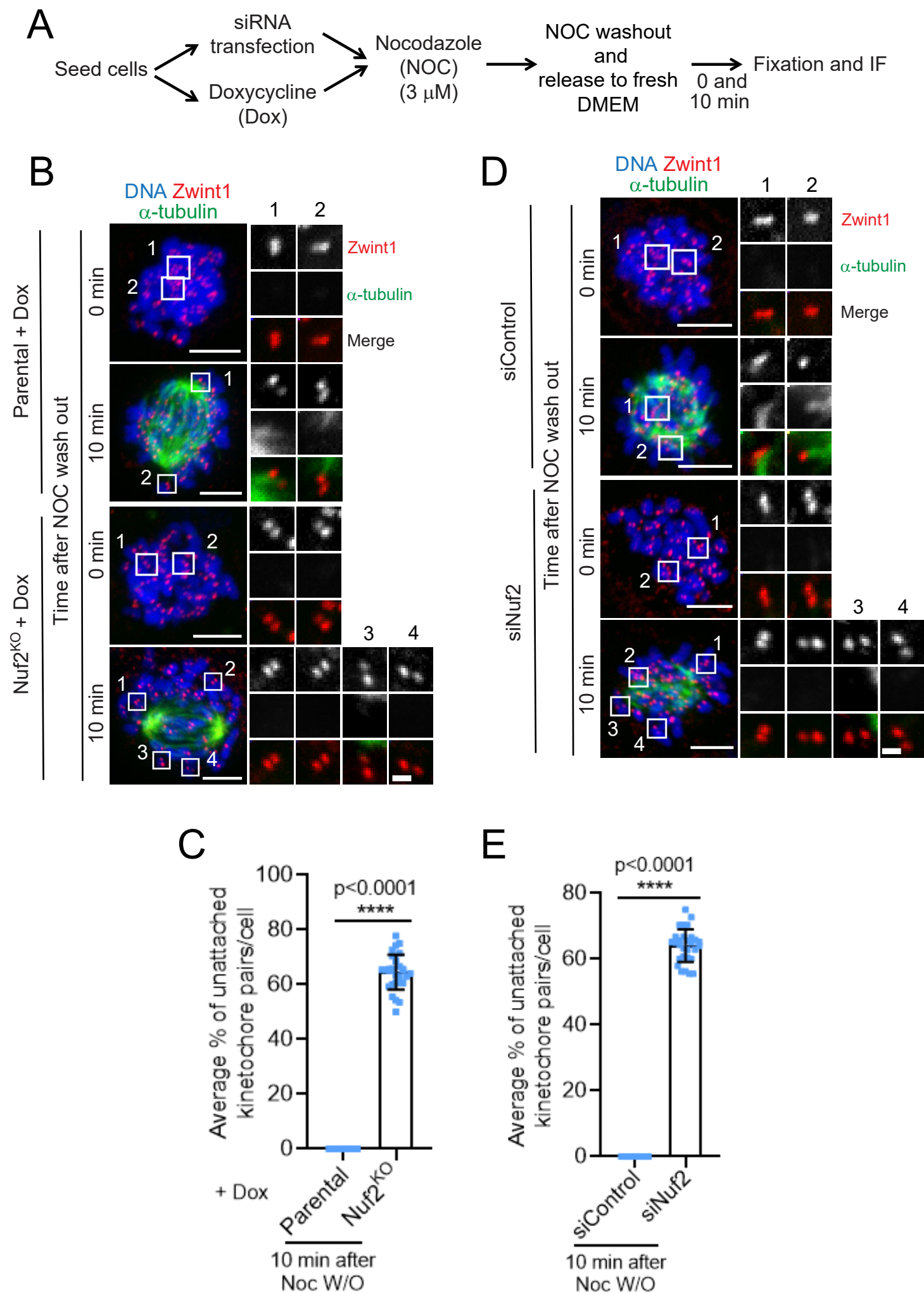

Supplement: Supporting Figure S3 — Nocodazole wash out assay to assess kinetochore capture by de novo microtubules early in mitosis after different modes of Ndc80 inhibition.A, Cells were subjected to the indicated perturbation, treated with the indicated drug, and fixed according to the scheme. B and D, Immunofluorescence staining of mitotic HeLa cells knocked out of Nuf2 (B) or of mitotic RPE1 cells depleted of Nuf2 (D) in comparison to control or parental cells respectively and immunostained for α-tubulin (green), a kinetochore marker Zwint1 (red) with the chromosomes counterstained using DAPI. Bars, 5 μm. Inset shows the kMT attachment status of individual kinetochore pairs in the indicated conditions. Bar (applies to all insets), 1 μm. C and E, Quantification of the status of kMT attachments in cells from B and D. Error bars represent S.D. from three independent experiments. For each experiment, ten mitotic cells were examined. ∗∗∗∗P < 0.0001 (Student’s t test). [file mmc16.pdf]

Figure S4

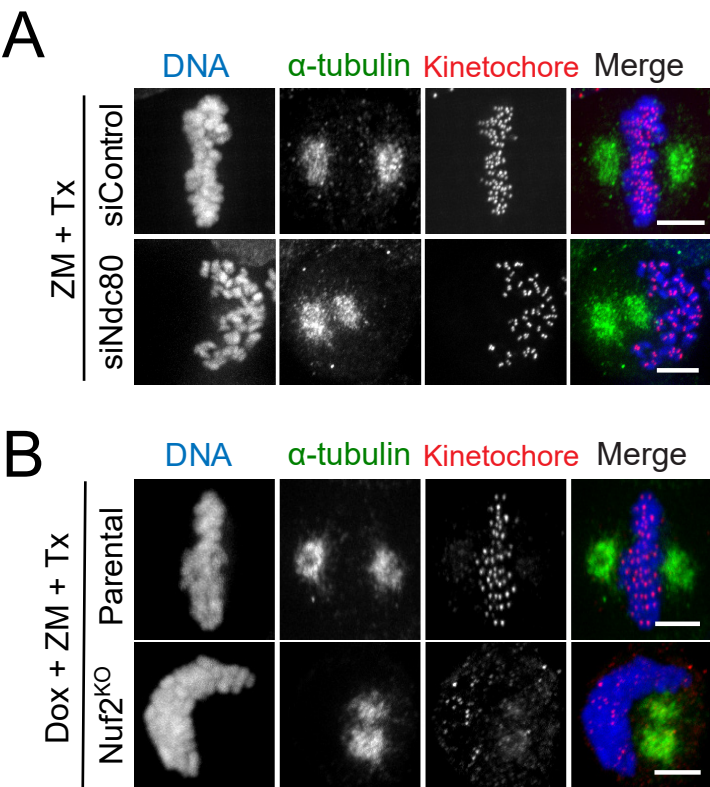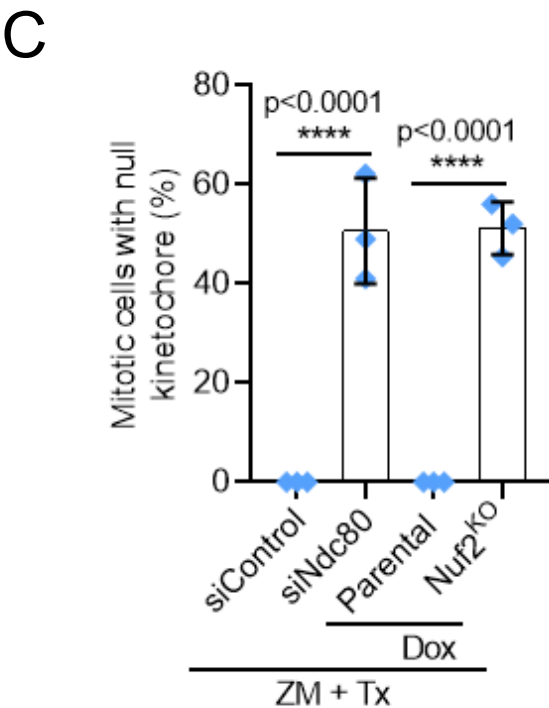

Supplement: Supporting Figure S4 — Further characterization of the kinetochore null phenotype and dynein kinetochore localization in Ndc80-inhibited cells.A and B, Mitotic HeLa cells depleted of Ndc80 (A, bottom panel) or knocked out of Nuf2 (B, bottom panel) as compared to controlRNAi (A, top panel) or parental control cells (B, bottom panel), and followed by the indicated drug treatments, were immunostained for α-tubulin (green), a kinetochore marker Zwint1 or CENPA (red) with the chromosomes counterstained using DAPI. Bars, 5 μm. C, Quantification of the frequency of mitotic cells with null kinetochores (see main text for more details) in samples from A and B. Error bars represent S.D. from three independent experiments. For each experiment, 200 mitotic cells were examined. ∗∗∗∗P < 0.0001 (Student’s t test). [file mmc17.pdf]

Figure S5

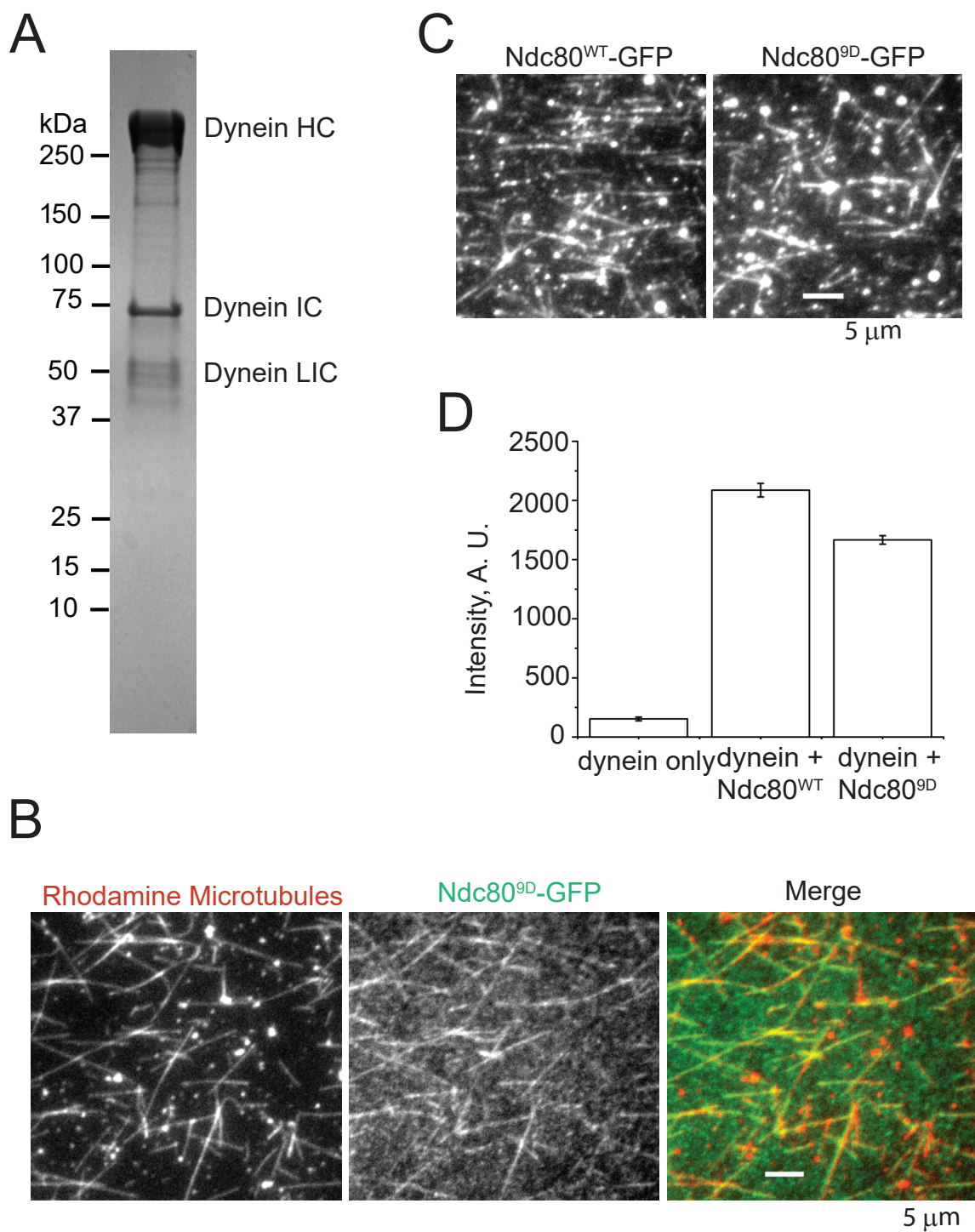

Supplement: Supporting Figure S5 — Ndc809Dmutant captures/binds microtubules in both the TIR-FM geometries tested.A, An SDS-PAGE electrophoretic gel of purified human whole cytoplasmic dynein complex used in the study. B, Images showing binding of Ndc809D bonsai-GFP to Taxol-stabilized Rhodamine-labeled microtubules immobilized on coverslips. Scale bar, 5 μm. C, Capture of microtubules by Ndc80wt or Ndc809D-bonsai in the geometry used for the microtubule-landing experiments as in Figure 6 where these proteins were immobilized on to coverslips and Rhodamine microtubules were floated-in. Scale bar, 5 μm. D, Plot of mean surface intensity of GFP-tagged Ndc80 proteins. The ‘dynein only’ measurements indicate the background. N = 3 independent trials. [file mmc18.pdf]

Figure S6

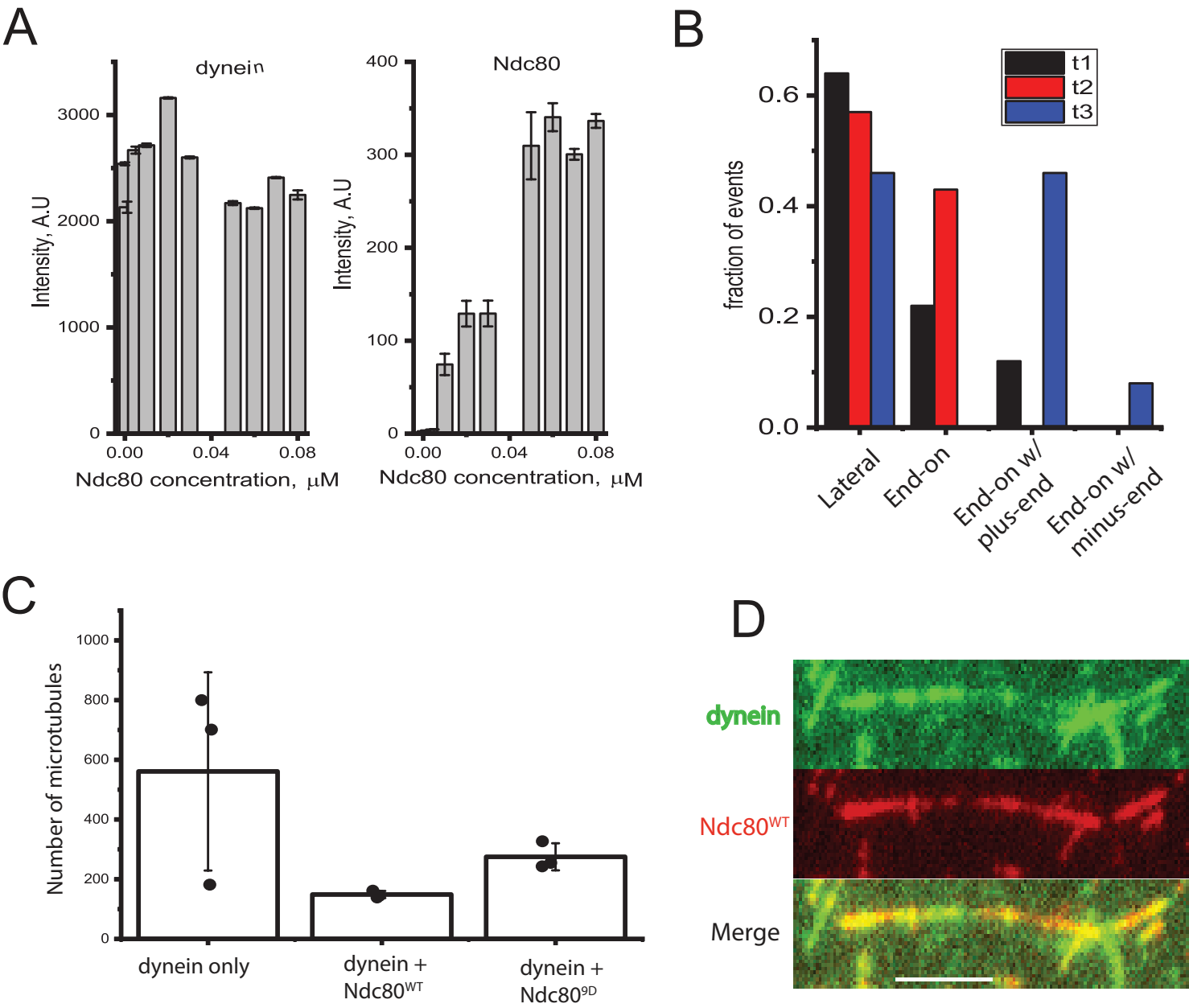

Supplement: Supporting Figure S6 — Characterization of Dynein Ndc80 interaction using multiple parameters.A, Plot of mean surface density of dyneinalexa488(left), and Ndc80alexa647(right) as a function of surface Ndc80alexa647 concentration. Data is Mean ± SEM. B, Fraction of the various kinds of microtubules landing behavior with dynein plus Ndc80 (consolidated numbers for Ndc80 9D and wt-bonsai, B and C from above) immobilized on the surface. t1, t2, and t3 indicate different trials on different days. C, Quantification of the number of microtubules bound to the surface for indicated proteins. Bars, mean ± SD. D, Montage showing Dyneinalexa488 (top, concentration 50 nM), Ndc80alexa647 (center, concentration 50 nM) and merge (bottom) bound to taxol stabilized microtubule (not imaged). Bar is 5 μm. [file mmc19.pdf]
